# Complement and neutrophils are actively involved at the cervicovaginal interface in cases of adverse microbial composition, cervical shortening and spontaneous preterm birth

**DOI:** 10.1038/s41522-026-00969-x

**Published:** 2026-04-15

**Authors:** Belen Gimeno-Molina, Gonçalo dos Santos Correia, Sherrianne Ng, Yun S. Lee, Erna Bayar, Ryan L. Love, Sihao Zhao, Katherine E. Mountain, TG Teoh, Richard G. Brown, Anna L. David, Holly V. Lewis, Vasso Terzidou, Marina Botto, Pascale Kropf, David A. MacIntyre, Phillip R. Bennett, Lynne Sykes

**Affiliations:** 1 Imperial College Parturition Research Group, Institute of Reproductive and Developmental Biology, Department of Metabolism, Digestion and Reproduction, Imperial College London, London, UK; 2 March of Dimes Prematurity Research Centre at Imperial College London, London, UK; 3 Parasol Foundation Centre for Women’s Health and Cancer Research, St Mary’s Hospital, Imperial College Healthcare NHS Trust, London, UK; 4 KK Women’s and Children’s Hospital, Singapore, Singapore; 5 Elizabeth Garrett Anderson Institute for Women’s Health, University College London, London, UK; 6 Department of Obstetrics and Gynaecology, University of Hong Kong SAR, Hong Kong, China; 7 Chelsea and Westminster Hospital NHS Foundation Trust, London, UK; 8 Department of Immunology and Inflammation, Imperial College London, London, UK; 9 Department of Infectious Diseases, Imperial College London, London, UK; 10 Robinson Research Institute, University of Adelaide, Adelaide, Australia

**Keywords:** Diseases, Immunology, Medical research, Microbiology

## Abstract

Microbial-driven spontaneous preterm birth (sPTB) is linked to adverse vaginal microbiome and dysregulated immune responses, yet this knowledge has not translated into predictive tools or therapies. We sampled 186 high-risk pregnant women and assessed 14 complement proteins and the neutrophil immunophenotype at the cervicovaginal interface to expand potential targets. Alterations in classical and alternative complement pathway components were associated with bacterial community composition and preceded cervical shortening and sPTB. At 12^+0^–16^+6^ weeks, women with Community State Type (CST) IV had significantly higher concentrations of C1q, C4, C4b, Factor B, D, C3b/iC3b, and Factor H, I than those with CST I. Women who later developed a short cervix and delivered preterm showed lower *L. crispatus* abundance and elevated complement activation. Neutrophils were the dominant local immune cell and exhibited enhanced activation relative to peripheral neutrophils, with altered expression of CD11b, CD62L, CD63 and CD66b. Cervical neutrophil CD63, CD66b, and CD88 differed between CST IV and CST I, though not by pregnancy outcome. Neutrophil abundance correlated with cytokines, complement proteins, and MMPs, suggesting roles in inflammation and tissue remodelling. These findings highlight a microbiota-driven complement–neutrophil axis present before cervical remodelling and sPTB, identifying potential complement-based predictors and therapeutic targets.

## Introduction

Preterm birth (PTB), defined as birth occurring prior to 37 weeks of pregnancy, is the leading cause of mortality and morbidity in children under the age of five^1,2^. PTB can be classed as either iatrogenic (medically indicated due to maternal or foetal indications) or spontaneous (sPTB), the latter accounting for two-thirds of cases^3^. Global rates of PTB have remained unchanged over the last decade, with 13.8 and 13.4 million babies being born premature in 2010 and 2020, respectively, accounting for almost 10% of all live births^4,5^. The development of improved prediction and new effective therapies requires a better understanding of the complex pathophysiology underlying the multiple different aetiologies of sPTB.

The past decade has highlighted the importance of the vaginal microbiome in driving sPTB. A vaginal microbial composition dominated by *Lactobacillus* spp., particularly *L. crispatus* (Community State Type I, CST I), has consistently been associated with optimal pregnancy outcomes and term birth; whereas women with a sub-optimal microbiome characterised by *L. iners* dominance (CST III) or depletion of *Lactobacillus* spp. with increased diversity (CST IV) are at higher risk of cervical shortening^6–8^ and sPTB^8–15^. An increase in concentrations of pro-inflammatory mediators, particularly cytokines and chemokines, in various body fluids, including peripheral blood, cervicovaginal and amniotic fluid, has also been described in association with sPTB^16–18^. Since the introduction of next-generation sequencing, complex associations between pro-inflammatory mediators and adverse vaginal bacterial community composition have also been described^10,15,19,20^.

The complement system is formed by a large number of proteins that assist with the recognition and clearance of microbes, which can be activated via the classical pathway, the lectin pathway and/or the alternative pathway. All three pathways converge in the activation of C3 convertases, which release C3a, a potent anaphylatoxin that recruits neutrophils, and C3b, which contributes to the self-amplification loop and opsonisation to facilitate phagocytosis. Activation of C5 convertase leads to the release of anaphylatoxin C5a and C5b, necessary for the creation of the membrane attack complex (MAC)^21^. While the importance of the complement system in regulating pregnancy has been recognised^21,22^, human studies reporting on the potential role of complement proteins in PTB are mainly limited to describing very few proteins and at sites distant to the cervicovaginal interface, such as fragment Bb in peripheral blood^23,24^ or C3a, C4a and C5a in amniotic fluid^25–27^. Despite this, there is compelling evidence of the role of complement in microbial-driven preterm birth in mice. Lipopolysaccharide (LPS) induced PTB in mice is associated with C3 deposition in the cervix and increased serum concentrations of C3a and C5a^28^, and C5a receptor knockout mice are protected from LPS-induced cervical remodelling and PTB^28^. Our group has previously demonstrated increasing concentrations of a limited array of complement proteins, MBL, C3b/iC3b and C5a, in the cervicovaginal fluid (CVF) of women with sub-optimal vaginal microbiomes who deliver spontaneously preterm^15^. However, there is a much larger array of complement proteins and regulators that may be implicated in response to adverse microbial composition, offering a broader set of potential novel predictive and therapeutic targets.

Activation of the complement system facilitates the recruitment and activation of phagocytes such as neutrophils^29,30^. Pregnancy is characterised by a peripheral blood neutrophilia^31–36^ and neutrophil activation^33,36,37^. Neutrophil migration to the cervicovaginal interface has been detected by immunohistochemistry of in-labour tissue biopsy samples^38,39^, and by flow cytometry analysis of cytobrush samples^40^. Transcriptomic examination has linked genes associated with neutrophil activation to vaginal microbiota profiles^41^. Collectively, these studies suggest that cervical neutrophils influence host-microbe interactions and cervical remodelling^39,42^, and, therefore, may play a role in microbial-driven cervical shortening and sPTB. However, the immunophenotype of cervical neutrophils remains undescribed in the context of microbial composition, subsequent cervical shortening and/or sPTB.

Here, we report the comprehensive assessment of 14 complement proteins measured in CVF and detailed characterisation of the cervical neutrophil immunophenotype in relation to vaginal bacterial communities, cervical shortening, and sPTB. These findings can be exploited to develop new predictive biomarkers and therapeutic strategies for microbial and inflammation-driven sPTB.

## Results

### Study cohort and design

**Fig. 1:**
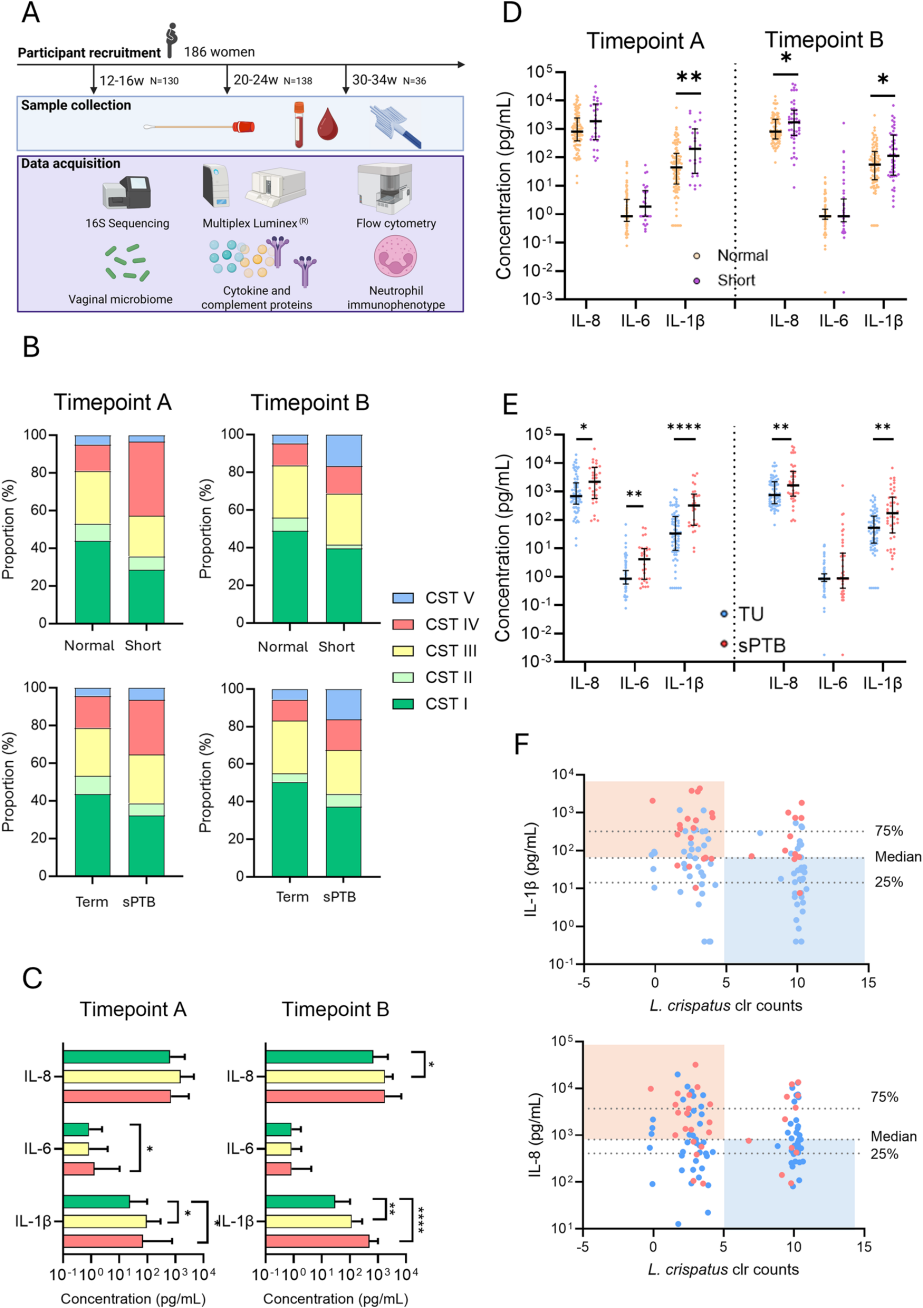
Study design, vaginal microbial composition and cervicovaginal fluid cytokines concentrations. **A**. 186 women provided 130 samples at timepoint A (12^+0^–16 + 6 weeks), 138 samples at timepoint B (20^+0^–24^+6^ weeks of gestation) and 36 samples at timepoint C (30^+0^–34^+6^ weeks of gestation). Samples included vaginal swabs, peripheral blood and cytobrush samples for 16S sequencing, Luminex immunoassays and flow cytometry. **B** Stacked bars depicting proportions of CSTs (Community State Types) in women with normal (timepoint A, *n* = 100; timepoint B, *n* = 86) and short cervix (timepoint A, *n* = 28; timepoint B, *n* = 48) and in women who delivered at term (timepoint A, *n* = 94; timepoint B, *n* = 89) or spontaneously preterm (timepoint A, *n* = 31; timepoint B, *n* = 43). Chi-square test was used to compare groups. **C** Concentration of IL-8, IL-6 and IL-1β (all pg/mL) according to microbial composition. Comparisons were made between CST I (*L. crispatus*) (A, *n* = 52; B, *n* = 61), III (*L. iners*) (A, *n* = 34; B, *n* = 37), IV (diverse) (A, *n* = 22; B, *n* = 17) using Kruskal–Wallis with post-Dunn’s test. **D** Concentration of cervicovaginal cytokines according to cervical length (normal = >25 mm (orange) short = ≤25 mm (purple)) and **E** pregnancy outcome (term uncomplicated TU (blue) vs spontaneous preterm birth sPTB (red)). Mann–Whitney two-tailed test was used for comparisons. **F** *L. crispatus* relative abundance and local inflammation**;** depiction of the correlation between the centred log-ratio (clr) counts of *L. crispatus* and the concentration of IL-8 and IL-1β, according to pregnancy outcome. Timepoint A = 12^+0^–16^+6^ weeks of gestation and timepoint B = 20^+0^–24^+6^ weeks of gestation. CST I: *L. crispatus*, II: *L. gasseri*, III: *L. iners*, IV: diverse, V: *L. jensenii*. *P* value summary: *p < 0.05; **p < 0.01; ***p < 0.001; ****p < 0.0001.

A total of 304 samples were collected from 186 pregnant women at high risk of preterm birth over three cross-sectional sampling points: timepoint A (12^+0^–16^+6^ weeks) *n* = 130, timepoint B (20^+0^–24^+6^ weeks) *n* = 138, and timepoint C (30^+0^–34^+6^ weeks) *n* = 36 (Fig. 1A). Matching samples (*n* = 296) for microbiome and immune mediator analyses were collected from 180 women, whilst 79 women also provided matched blood and cytobrush samples (*n* = 122) for peripheral and cervical neutrophil analyses (Fig. S1A). A subset of women had longitudinal samples collected for microbiome and immune mediator studies (Fig. S1B). Cervical shortening (cervical length ≤25 mm) and pregnancy outcome (sPTB <37 weeks) were independently evaluated (Fig. S1C). A total of 58 women had sPTB, 36/58 (62%) were classified as late preterm (34^+0^–36^+6^) and 22/58 (38%) early preterm (<34^+0^) (Table S1). Women with medically indicated PTB were excluded from the study. Cervical shortening occurred in 66/186 women (35.5%) (Table S2). Most women who delivered sPTB had intervention with cerclage and/or vaginal progesterone (39/58 women, 67.4%). Of the women who delivered at term, 44/124 (35.5%) received an intervention with cervical cerclage and/or progesterone (term intervention, TI) and 80/124 (64.5%) did not receive any treatment (term uncomplicated, TU) (Table S1). There were no statistically significant differences in BMI (*p* = 0.4216) or ethnicity (*p* = 0.0514) between women who delivered spontaneously preterm or at term with or without intervention, although women who delivered sPTB were slightly younger (*p* = 0.0014) (Table S1).

### Vaginal bacterial communities dominated by *Lactobacillus crispatus* associate with lower cervicovaginal cytokine concentrations, normal cervical length and term birth

Metataxonomic profiling of vaginal bacteria was performed on 298 swabs, generating 8,926,865 high-quality reads with an average read count of 29,956 per sample. The majority of the cohort was dominated by *L. crispatus, L. iners* or *G. vaginalis* (Fig. S2A). Samples were grouped into five bacterial community state types (Fig. S2B) using the VALENCIA classifier. Four were dominated by *Lactobacillus* spp.: CST I- *L. crispatus* (43.6%*)*, CST II- *L. gasseri* (7.4%), CST III -*L. iners* (25.8%), CST V -*L. jensenii* (7%). The CST IV (16.1%)- diverse and bacterial vaginosis-like profile could further be sub-classified into CST IV-A (1/48, 2.1%) with a high abundance of *Candidatus Lachnocurva vaginae*/BVAB1; CST IV-B (36/48, 75%) with a high relative abundance of *G. vaginalis* or IV-C (11/48, 23%). Both alpha diversity (Shannon index) and richness (number of species observed) were higher in microbiomes assigned as CST IV compared to CST I and CST III (*p* < 0.0001) (Fig. S2C). The Shannon index was also increased in women who developed cervical shortening and in women who delivered spontaneously preterm (Fig. S2D). A non-significant trend was observed between vaginal microbial composition and pregnancy outcome. Women who maintained a normal cervical length and who delivered at term had a higher prevalence of CST I; whereas women with a short cervix or who delivered spontaneously preterm had higher CST IV prevalence (Fig. 1B).

Sub-optimal microbiome compositions (CST III and CST IV) were associated with increased CVF cytokine concentrations. As early as 12^+0^–16^+6^ weeks (timepoint A), we detected increased IL-6 (CST I vs CST IV, *p* = 0.0386) and IL-1β (CST I vs CST III, *p* = 0.0284; CST I vs CST IV, *p* = 0.0119) (Fig. 1C and Table S3). Higher cytokine median concentrations were also observed in women who developed a short cervix compared to those who maintained a normal cervical length (Fig. 1D) (Timepoint A, IL-1β, *p* = 0.0029). Additionally, median concentrations of IL-8 and IL-1β were significantly higher in women who went on to deliver spontaneously preterm compared to those who delivered at term at both A and B timepoints (IL-8, *p* = 0.0103 and *p* = 0.0083; IL-1β, *p* < 0.0001 and *p* = 0.0013, respectively) (Fig. 1E). We next represented dot plots for investigating the interplay between the relative abundance of *L. crispatus*, cytokine concentrations and clinical outcomes. There was a significant negative correlation between the abundance of *L. crispatus* and the concentration of IL-8 (timepoint B, Spearman ρ = −0.1786, *p* = 0.0397) and IL-1β (timepoint A, Spearman ρ = −0.335, *p* = 0.0001; B, Spearman ρ = −0.417, *p* < 0.0001) (Table S4). Although more women who experienced cervical shortening clustered in the top left quadrant (lower *L. crispatus* centred log-ratio (clr) counts and higher concentrations of CVF cytokines), this did not reach statistical significance (Fig. 1F and Table S4). A significant negative correlation was seen between the abundance of *L. crispatus* and the concentration of IL-1β (timepoint A, Spearman ρ = −0.2905, *p* = 0.0031), and women who delivered spontaneously preterm were more likely to have lower relative abundance of *L. crispatus* and higher concentrations of IL-8 (*p* = 0.0106) and IL-1β (*p* = 0.0377).

### Complement associates strongly with *L. crispatus* depletion, high diversity communities, short cervix and spontaneous preterm birth

**Fig. 2:**
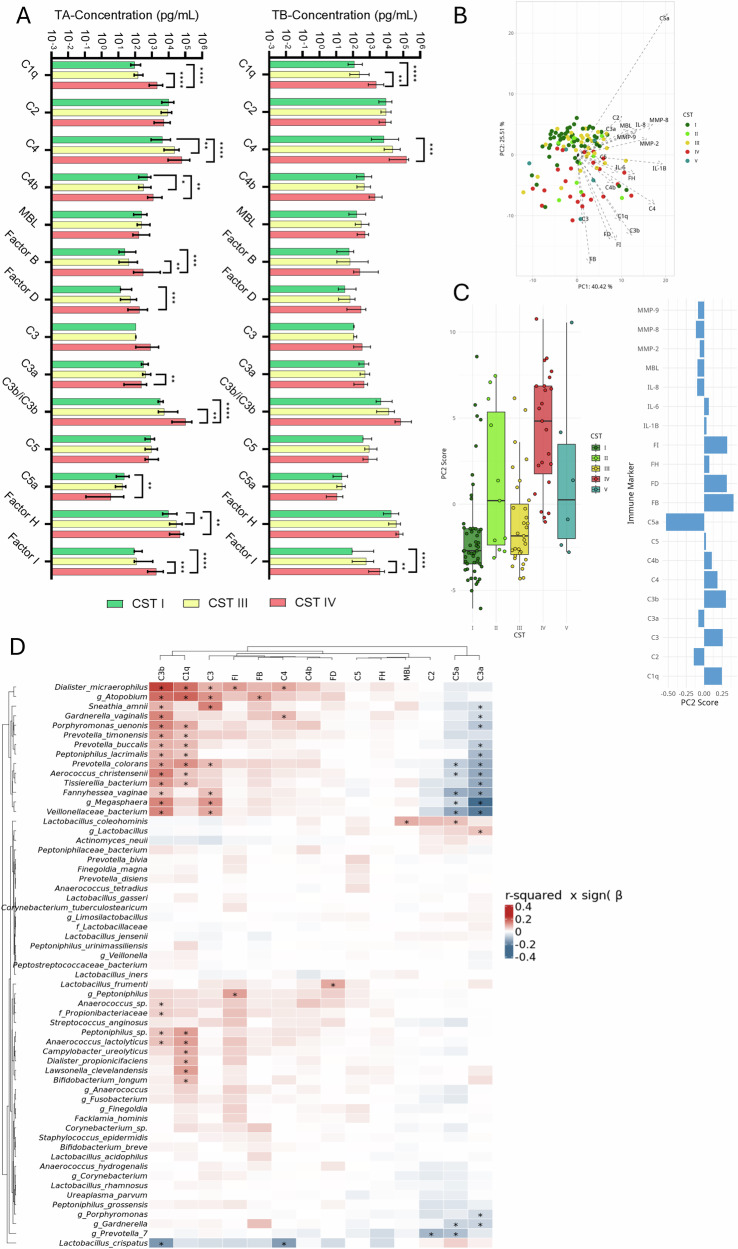
Cervicovaginal fluid complement protein concentrations according to microbial composition. **A** Median and interquartile range concentrations (all pg/mL) of C1q, C2, C4, C4b, MBL, Factor B, Factor D, C3, C3a, C3b/iC3b, C5, C5a, Factor H and Factor I in women with CST I (*L. crispatus*), III (*L. iners*) and IV (diverse) at timepoint A (TA) (12^+0^–16^+6^ weeks of gestation) (*n* = 108) and timepoint B (TB) (20^+0^–24^+6^ weeks of gestation) (*n* = 114). Differences between CSTs were assessed with Kruskal–Wallis with post-Dunn’s test. **B** PCA biplot with observations coloured by CST. **C** Box-and-whiskers plot of principal component 2 scores and their distribution between different levels of CST. Bar plot of the loading vector values for principal component 2. **D** Heatmap showing the association (regression model *r*^2^ with the linear regression coefficient sign) between key bacterial taxa and complement cascade protein concentrations, estimated with pairwise linear regression analyses. Hierarchical clustering analysis and dendrograms of bacterial taxa (rows) and complement proteins (columns) were obtained using Euclidean distance and complete linkage. Cells marked with * remain significant after Benjamini–Hochberg false discovery rate correction (*q* value <0.05).

At timepoint A, higher median concentrations of many complement proteins were observed in women with CST IV compared to women with CST I of complement proteins, including C1q (2067 vs 9478 pg/mL, *p* < 0.0001), C4 (63751 vs 4203 pg/mL, *p* < 0.0001), C4b (1218 vs 547.9 pg/mL, *p* = 0.0154), Factor B (301.8 vs 24.88 pg/mL, *p* = 0.0004), Factor D (185.1 vs 13.5 pg/mL, *p* = 0.0001), C3b/iC3b (101847 vs 3132 pg/mL, *p* < 0.0001), and complement regulators Factor H (48297 vs 10090 pg/mL, *p* = 0.0077) and I (1864 vs 90 pg/mL, *p* < 0.0001) (Fig. 2A and Table S3). At timepoint B, although C1q, Factor B, Factor D, C4, C4b, C3b/iC3b, Factor H and Factor I concentrations appeared significantly different according to microbial composition, only C1q, C4b and Factor I differences persisted after multiple comparisons correction (Table S3).

Complement proteins were also significantly increased in CST III compared to CST I (at timepoint A: C4, *p* = 0.0032; Factor H, *p* = 0.0228), although some complement protein median concentrations were lower in women with CST III compared to CST IV (C1q 147.6 vs 2067 pg/mL, *p* < 0.0001), Factor B (42.7 vs 301.8 pg/mL, *p* = 0.0016), C3b/iC3b (5594 vs 101847 pg/mL, *p* = 0.0016), Factor I (121.5 vs 1864 pg/mL, *p* = 0.0006). Lower median concentrations of C3a were detected in CST IV compared to CST III (237 vs 395.1 pg/mL, *p* = 0.0041); and lower C5a between CST IV (3.64 pg/mL) compared to CST I (22.31 pg/mL, *p* = 0.0039).

Principal component analysis (PCA) of complement proteins revealed co-clustering patterns that were seemingly driven by microbiota composition (Fig. 2B). PC1 and PC2 accounted for 40 and 25% of the total variance, respectively, with separation largely driven by microbial composition, especially alongside PC2 (Fig. 2C). This component is predominantly associated with covariation in C5a, Factor B, Factor D, Factor I, C1q and C3b/iC3b (Fig. 2C), suggesting a key role of complement activation in response to *Lactobacillus* depleted communities. Strong positive correlations were observed between all immune mediators with each other, regardless of microbial composition (Fig. S3).

To identify associations between immune mediator concentrations and relative abundance of specific bacterial taxa, multiple independent linear regression analyses were performed and presented in a heatmap (Fig. 2D). *L. crispatus* negatively correlated with pro-inflammatory immune mediators, whereas CST IV-type bacteria (e.g. *Gardnerella vaginalis, Sneathia, Prevotella, Atopobium*) are strongly correlated with C1q, C4, C3, C3b/iC3b, Factor I, and B (Fig. 2D), implicating the classical and alternative pathways. Interestingly, there was no strong association between the presence of *L. iners* and complement activation, and cervicovaginal supernatant concentrations of C5a and C3a were negatively correlated with some of the bacterial species typically clustered in CST IV.

**Fig. 3:**
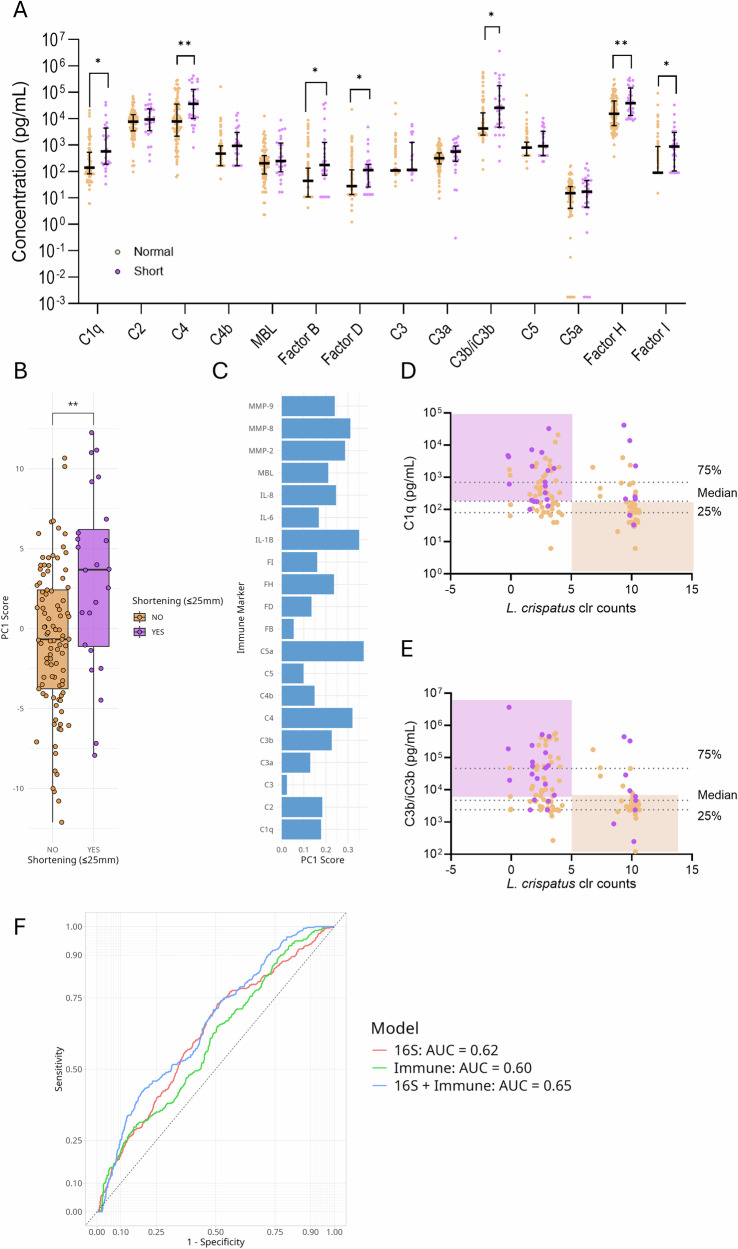
Cervicovaginal fluid complement protein concentrations according to cervical length. **A** Median and interquartile range concentrations of C1q, C2, C4, C4b, MBL, Factor B, Factor D, C3, C3a, C3b/iC3b, C5, C5a, Factor H and Factor I (all pg/mL) in women with normal (orange, >25 mm, *n* = 99) and short cervix (purple, ≤25 mm, *n* = 28). Differences were assessed with a two-tailed Mann–Whitney test. **B** Boxplot with PC1 scores and their association with cervical length. **C** Loading vector for PC1. **D**, **E** depicts the correlation between the clr-transformed counts of *L. crispatus* and the concentration of C1q and C3b/iC3b, according to cervical length (orange, normal; purple, short). **F** Area under the receiver-operating characteristics (ROC) curve (AUC) for the prediction of cervical shortening. All samples were collected at timepoint A (12^+0^–16^+6^ weeks of gestation). *P* value summary: *p < 0.05; **p < 0.01; ***p < 0.001.

Direct comparisons of cervicovaginal fluid complement concentrations were evaluated between women who had a normal length cervix and those who developed a short cervix at timepoint A (Fig. 3A) and at timepoint B (Fig. S4A and Table S5). Significantly increased median concentrations were seen for C1q (140 vs 576 pg/mL, *p* = 0.0452), C4 (7918 vs 36683 pg/mL, *p* = 0.0075), Factor B (43.89 vs 177.8 pg/mL, *p* = 0.0452), Factor D (27.98 vs 113 pg/mL, *p* = 0.0452),, C3b/C3bi (4262 vs 25734 pg/mL, *p* = 0.0452), Factor H (15432 vs 39006 pg/mL, *p* = 0.0039) and Factor I (90 vs 879.7 pg/mL, *p* = 0.0117). Statistical significance was also seen at timepoint B for C1q, C4, Factor B, C3a, C3b/iC3b, Factor H and Factor I (Fig. S4A and Table S5). A statistically significant association (*p* = 0.00264) was found between the development of cervical shortening and maintenance of a normal length cervix and the magnitude of local immune activation, as captured in PC1 (Fig. 3B). Complement proteins that provided the greatest weighting to these phenotypes included C5a, C4, C3b/iC3b, Factor H, C1q and C2. The cytokines IL-1β and IL-8, and the MMPs -2, -8, -9 also demonstrated comparable weightings (Fig. 3C). There was a significant negative correlation between *L. crispatus* clr counts and the concentration of complement proteins (Table S4), particularly C1q (Spearman ρ = −0.4285) and C3b/iC3b (Spearman ρ = -0.4551) (both *p* < 0.0001) (Fig. 3D, E). The interplay between microbial composition, complement proteins, and cervical shortening revealed that women with less relative abundance of *L. crispatus* and higher median concentrations of complement proteins were more likely to develop a short cervix, mostly with higher concentrations of C1q (*p* = 0.0374), C4 (*p* = 0.0442) and C3b (*p* = 0.0262) (Table S4). We present predictive models of cervical shortening, based on timepoint A samples as the most clinically relevant timepoint. Vaginal microbial composition (16S data) demonstrated an area under the curve (AUC) of 0.62; immune data (concentration of cytokines, complement proteins and MMPs) demonstrated an AUC of 0.60. The combination of both data sets (16S data and immune data) improved the diagnostic performance (AUC 0.65) (Fig. 3F).

**Fig. 4:**
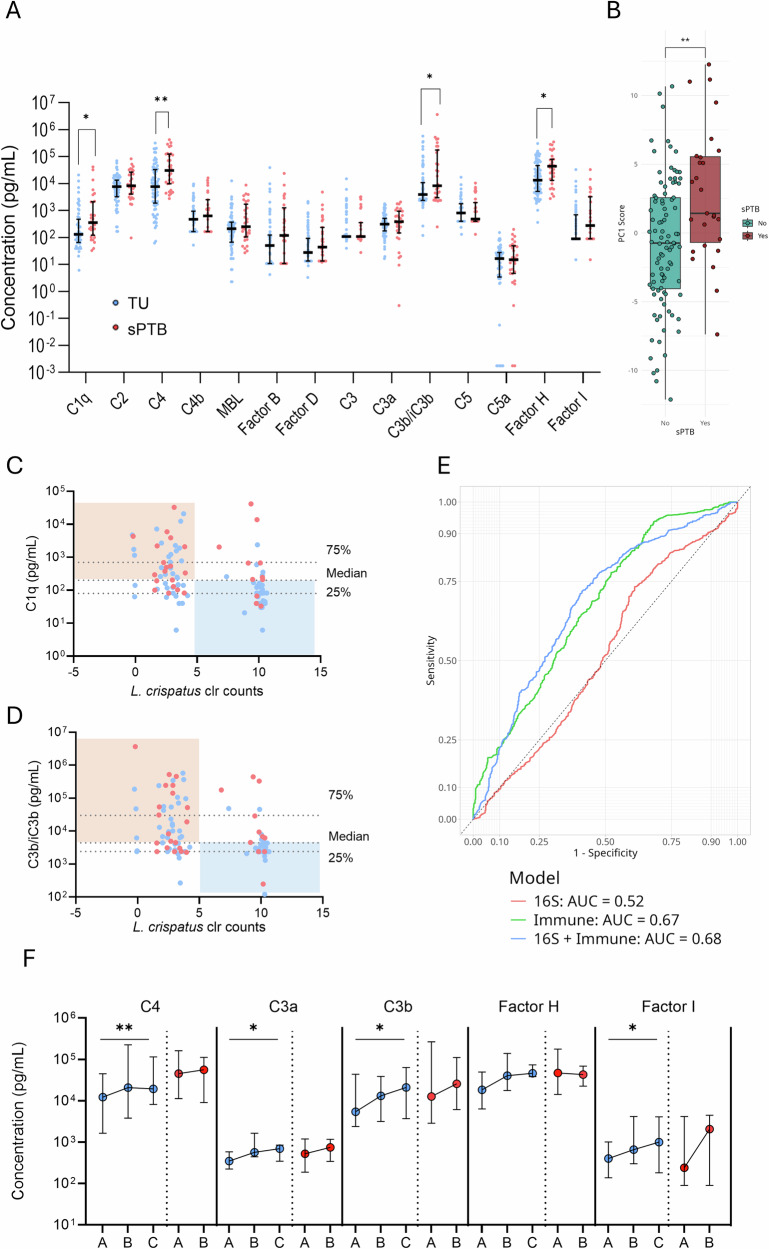
Cervicovaginal fluid complement protein concentrations according to pregnancy outcome. **A** Median and interquartile range concentrations of C1q, C2, C4, C4b, MBL, Factor B, Factor D, C3, C3a, C3b/iC3b, C5, C5a, Factor H and Factor I (all pg/mL) in women with term uncomplicated (blue, *n* = 74)) and spontaneous preterm birth (red, *n* = 31). Differences were assessed with two-tailed Mann–Whitney test. **B** Boxplot with PC1 scores their distribution according to pregnancy outcome. **C**, **D** depicts the correlation between clr-transformed counts of *L. crispatus* and the concentration of C1q and C3b/iC3b, according to pregnancy outcome (Term uncomplicated (TU), blue; spontaneous preterm birth (PT), red). All samples collected at timepoint A (12^+0^–16^+6^ weeks of gestation). **E** Area under the receiver-operating characteristics (ROC) curve (AUC) for the prediction of preterm birth **F** Concentration of analytes in the longitudinal sub-cohort. Representation of median and interquartile range. A = Timepoint A (12^+0^–16^+6^ weeks), B = timepoint B (20^+0^–24^+6^), C= timepoint C (30^+0^–34^+6^) *P* value summary: *p < 0.05; **p < 0.01; ***p < 0.001.

Cervicovaginal fluid complement concentrations were also compared between women who went on to deliver at term compared to spontaneously preterm from samples taken at timepoint A (Fig. 4A, B, Fig. S4B, and Table S5). Significantly increased median concentrations were seen of C1q (132.7 vs 365.5 pg/mL, *p* = 0.04935), C4 (7780 vs 30496 pg/mL, *p* = 0.001974), C3b/C3bi (3922 vs 8257 pg/mL, *p* = 0.02254) and Factor H (13360 vs 44045 pg/mL, *p* = 0.04935) as early as timepoint A. Factor B (77 vs 188.3 pg/mL, *p* = 0.0064), Factor D (44.78 vs 153.6 pg/mL, *p* = 0.00966) and C3a (354.2 vs 677.1 pg/mL, *p* = 0.0263) were increased only at timepoint B. As with cervical length, the CVF immune signature contributes significantly to differences seen by the pregnancy outcomes of term and sPTB (*p* = 0.0141), as shown by the boxplot from an unsupervised PCA (Fig. 4B). There was also a significant negative correlation between *L. crispatus* clr counts and the concentration of complement proteins according to pregnancy outcome, including C1q (Spearman ρ = -0.4416, *p* < 0.0001) and C3b (Spearman ρ = -0.4245, *p* < 0.0001) (Fig. 4C, D) among others (Table S4). Women with lower *L. crispatus* relative abundance, and higher concentrations of complement proteins were more likely to deliver spontaneously preterm; whereas women with higher proportions of *L. crispatus* and lower concentrations of C1q (*p* = 0.0126), C4 (*p* = 0.0077) and C3b/iC3b (*p* = 0.0233) were more likely to deliver at term (Table S4). The ability to predict preterm birth was tested at timepoint A, and the combination of microbial composition dataset (16S) together with the immune dataset exhibited the strongest predictive performance, with and AUC of 0.68, outperforming each individual dataset (AUC 0.52 and 0.67, respectively) (Fig. 4E). A trajectory of increasing complement concentrations across pregnancy ahead of term labour was also seen, however these increases were seen at later gestational age timepoints compared to women who subsequently delivered preterm (Fig. 4F). There was an increase in the median concentration between matched timepoint A (12^+0^–16^+6^ weeks) and C (30^+0^–34^+6^) for C4 (12238 vs 19486 pg/mL, *p* = 0.0059), C3a (349.4 vs 693.7 pg/mL, *p* = 0.0273), C3b (5431 vs 21037 pg/mL, *p* = 0.0273), Factor H (18410 vs 4615 pg/mL, *p* = 0.1602) and Factor I (400.7 vs 1000 pg/mL, *p* = 0.0391) (Fig. 4E).

### Neutrophils are the dominant immune cell at the cervical interface, are more activated than peripheral blood neutrophils, and in association with a CST IV

**Fig. 5:**
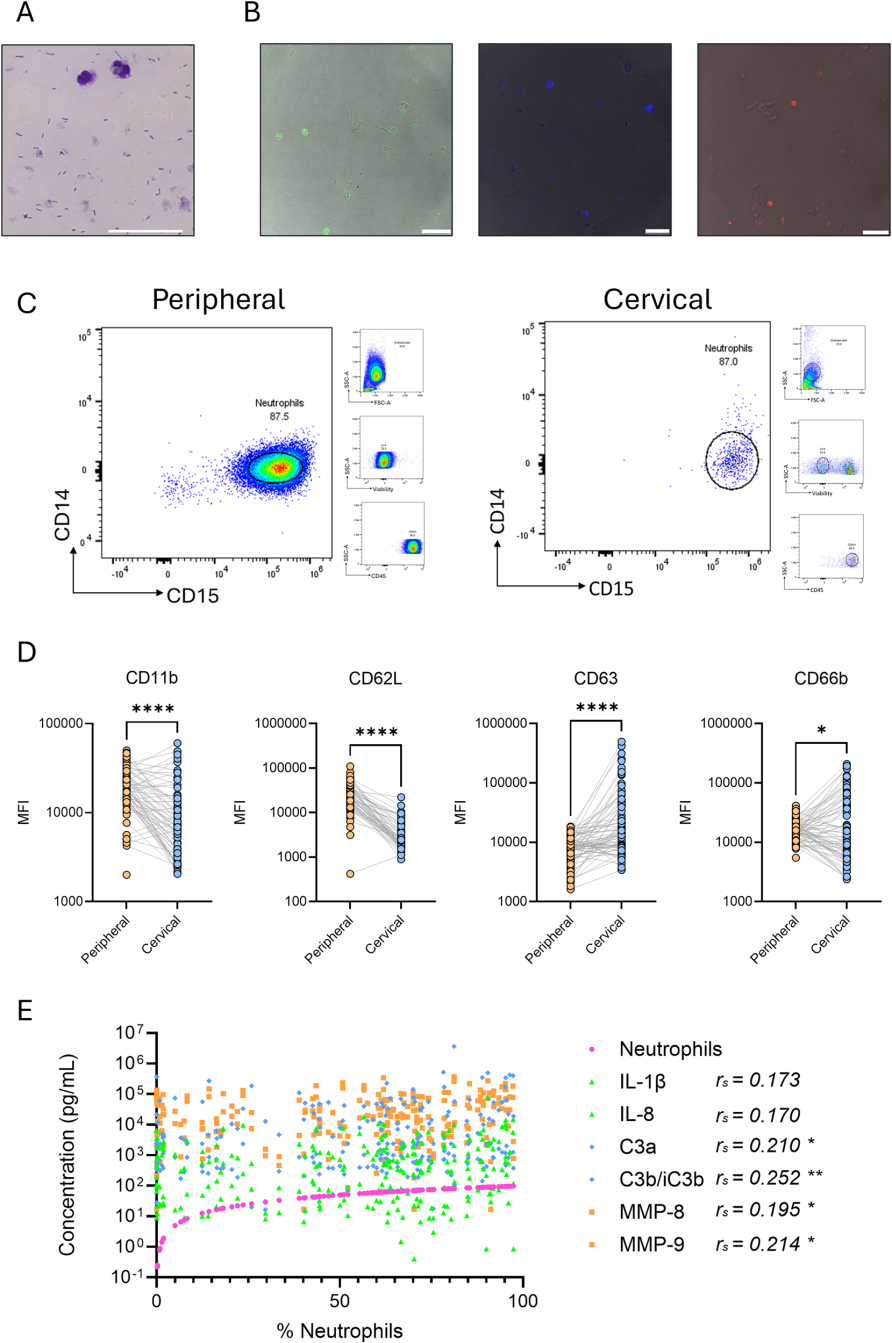
Description of neutrophils at the cervicovaginal interface. **A** Neutrophils were identified in cytobrush smears after Giemsa staining. Scale bar indicates 100 µm. **B** Neutrophils were identified by confocal microscopy after staining with CD15, CD16 and CD63, respectively. Scale bar indicates 50 µm. **C** Gating strategy for peripheral blood neutrophils and cervical neutrophils. **D** MFI (median fluorescence intensity) of neutrophil surface markers (CD11b (*n* = 68), CD62L (*n* = 57), CD63 (*n* = 70) and CD66b (*n* = 69)) between matched peripheral and cervical neutrophils by Wilcoxon matched-pairs signed rank test. **E** Spearman correlations between the percentage of cervical neutrophils and the concentration of IL-1β, IL-8, C3b/iC3b, C3a, MMP-8 and MMP-9 (*n* = 122). *P* value summary: *p < 0.05; **p < 0.01; ***p < 0.001; ****p < 0.0001.

Given the established interplay between complement and neutrophils, and the capacity of neutrophils to generate labour-associated proteins and pro-inflammatory mediators, we investigated the immune cell composition at the cervicovaginal interface. Cervical cytobrush samples were collected to determine the predominant immune cells at the cervicovaginal interface, and matched peripheral blood was collected to compare local and peripheral immune cell phenotypes. Figure 5A demonstrates the morphological appearance of neutrophils from a cytobrush sample after Giemsa staining. Cervical neutrophils demonstrate binding with the fluorescently labelled anti-CD15, anti- CD63 and anti-CD16 antibody (Fig. 5B). Cells were identified using flow cytometry (Fig. S5), with the neutrophil population detected as CD45^+^ and CD15^high^/CD14^low^ from both cytobrush and peripheral blood samples (Fig. 5C and S5A, B). Neutrophils were the dominant immune cell type of the cervical mucosal surface, especially in women of CST IV (Fig. S5C). Macrophages were identified as CD14^high^/ CD15^neg^ and detected in a smaller proportion of women (11%, 14/127), predominantly in CST I, and rarely detected in CST III and IV (Fig. S5C). Cervical neutrophils were found to be more activated than matched peripheral blood neutrophils, as determined by loss of expression of integrin CD11b (*p* < 0.0001) and selectin CD62L (*p* < 0.0001), and by increased expression of effector function marker CD63 (*p* < 0.0001) (Fig. 5D and Table S6A). Cervical neutrophil CD66b MFI was significantly higher compared to peripheral neutrophils in CST IV, whereas a reduction in expression was seen in women of CST I (*p* = 0.0083). There was a positive correlation between the percentage of cervical neutrophils and the concentration of IL-1β, IL-8, C3b/iC3b, C3a, MMP-8 and MMP-9 (Fig. 5E). Neutrophils are a source of MMPs and may contribute to tissue degradation and remodelling, since significantly higher concentrations were observed in women who experienced cervical shortening and who delivered spontaneously preterm (Fig. S6).

**Fig. 6:**
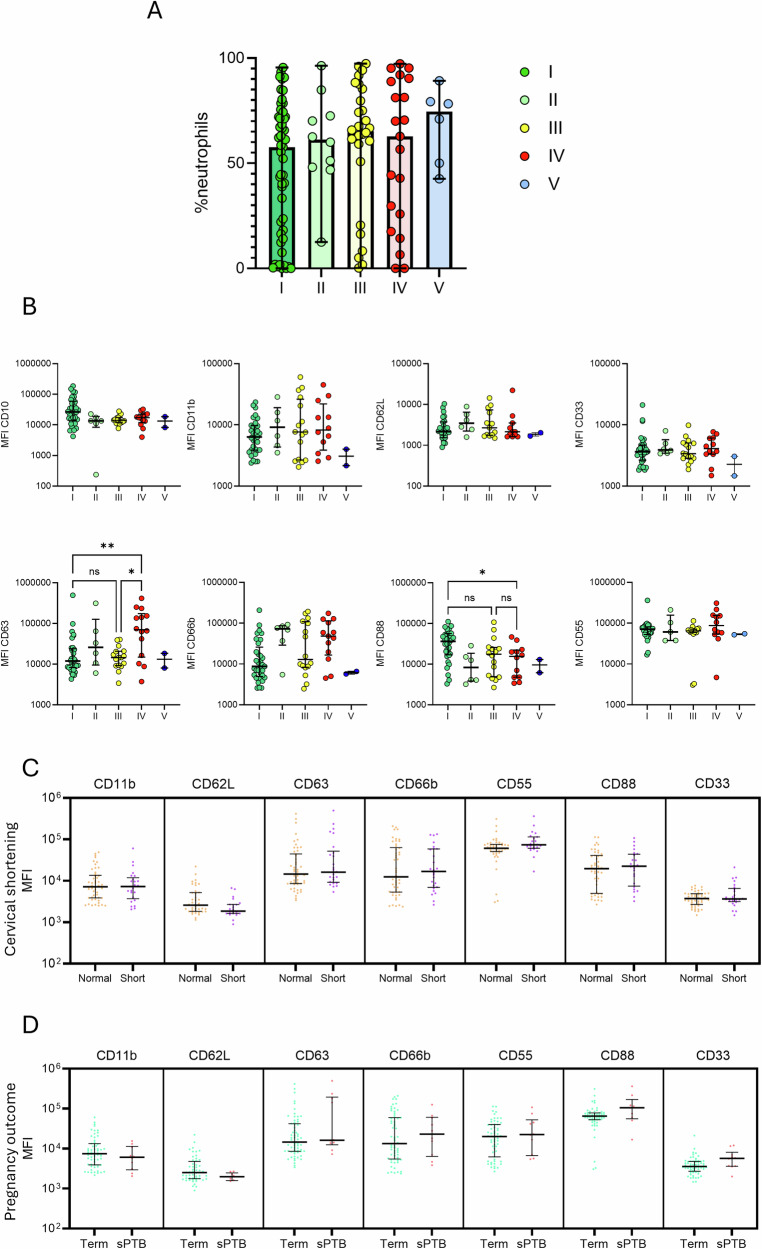
Characterisation of cervical neutrophils. **A** Percentage of cervical neutrophils according to microbial composition. Proportions were compared between community state (CST I, *n* = 51; CST III, *n* = 28; CST IV, *n* = 21) types using Kruskal–Wallis with post-Dunn’s correction. **B** MFI of cervical neutrophil surface markers (CD11b, CD62L, CD63, CD66b, CD35, CD55, CD88 and CD33, respectively) were compared according to microbial composition (CST I, *n* = 33; CST III, *n* = 16; CST IV, *n* = 13) using Kruskal–Wallis with post-Dunn’s correction. **C** MFI of neutrophil surface markers according to cervical shortening (normal, *n* = 50; short, *n* = 25) and **D** pregnancy outcome (term, *n* = 62; sPTB, *n* = 10). Comparisons were evaluated using Mann–Whitney two-tailed tests. *P* value summary: *p < 0.05; **p < 0.01.

Interestingly, all women had cervical neutrophils detected regardless of their CST composition (*p* = 0.2244) (Fig. 6A). However, there was a higher proportion of women with large proportions of neutrophils (≥50%) in CST III compared to CST I (*p* = 0.0325). Moreover, there were unique changes in the cell surface markers of cervical neutrophils according to CST (Fig. 6B), but a negligible effect on peripheral neutrophils (Table S6B). There was an increase in the expression of CD63 in cervical neutrophils of women with CST IV compared to CST I (*p* = 0.0039). A similar trend was observed for CD66b (*p* = 0.052). Furthermore, we found a reduction in the expression of CD88 (C5a receptor 1) between women of CST III and IV with women of CST I (*p* = 0.0231) (Fig. 6B). When comparing the expression of markers of activation with different pregnancy outcomes (Table S6C and Fig. 6C, D), there were no MFI patterns associated with cervical shortening or spontaneous preterm birth.

## Discussion

Many studies have reported associations between vaginal bacterial composition and the risk of cervical shortening and PTB^8,10,14,15,43,44^, information that has been used in machine learning approaches to predict preterm birth^45^. It is widely accepted that the cervicovaginal immune milieu plays a key role in driving microbial-induced preterm birth^15,16,18,46^. However, most studies have reported on a very limited array of immune mediators such as cytokines and chemokines, which restricts the development of predictive biomarkers and the potential to exploit immune modulation as a targeted therapeutic intervention for a precision medicine approach to the prevention of spontaneous preterm birth.

Uniquely, this study has comprehensively assessed 14 complement proteins in the CVF, implicating a key role of the complement cascade in microbial-driven spontaneous preterm birth. Activation of differential components from the classical and lectin pathways (C1q, MBL, C2, C4 and C4b), the alternative pathway (Factor B and Factor D), C3 convertase products (C3, C3a and C3b), C5 convertase products (C5 and C5a), and regulators (Factor H, Factor I) was associated with adverse vaginal microbial composition, subsequent cervical shortening and sPTB. Taken together, these data support the potential for clinical use of CVF complement proteins as predictive biomarkers for sPTB, and highlights the potential of repurposing complement-targeted therapeutics and developing of live biotherapeutics for sPTB prevention.

The classical pathway is activated via antigen-antibody complexes binding to C1q, inducing conformational changes of C1r into the active C1s. This serine protease cleaves C4 and C2. We detected increased C1q, C4 and C4b in association with CST IV, supporting the role of the classical pathway in responding to CST IV-type taxa. This is consistent with work by Livson et al., which detected cleavage of C4 by western blot in association with *G. vaginalis*^47^. The alternative pathway is maintained by spontaneous hydrolysis of C3, then binding of Factor B (FB). Factor D (FD) cleaves FB, generating the attached fragment Bb to form C3 convertase. Despite relatively low concentrations of both FB and FD in CVF, both factors were elevated in women with CST IV, indicating that the alternative pathway could contribute to in situ complement amplification. Livson et al. also detected Factor B in association with *Atopobium vaginae*^47^. C3 convertases cleave C3 and release C3a and C3b. We detected increased C3 and C3b proteins in association with most of the taxa contributing to CST IV. Pellis et al demonstrated deposition of C3 in clue cells and on *G. vaginalis* isolated from vaginal lavages, indicating C3 activation^48^. Factor H and Factor I are complement regulators that inhibit the amplification loop of the alternative pathway. Elevation of these proteins in CST IV-type taxa is suggestive of an attempt at complement immune autoregulation. We conclude that the immune response detected in CST IV women is potentially complement-driven. Importantly, our finding that *L. crispatus* dominance (CST I) is negatively correlated with CVF complement proteins and cytokines, particularly C3, C3b/iC3b, C4 and IL-1β, aligns with optimal vaginal health, and supports the potential role of *L. crispatus* live biotherapeutics for preterm birth prevention.

Despite demonstrating higher concentrations of the cytokines IL-8 and IL-1β in women of CST III/ *L. iners* dominance, there was no strong evidence for complement cascade activation. The impact of *L. iners* dominance in the context of vaginal health is conflicting in the literature with some populations having a higher risk of preterm birth, and others not^8,13,49^. *L. iners* differs from other lactobacilli by not releasing hydrogen peroxide and producing L-lactate rather than D-lactic acid^50–52^; it is also associated with reduced community stability, often transitioning to other CSTs^13,53^. It seems likely, therefore, that cervical shortening and sPTB are triggered by differing immune mechanisms in women with vaginal CST III/ *L. iners* dominance or CST IV. It is possible that the inflammatory response seen in CST III is neutrophil-driven, where a high median proportion of cervical neutrophils was reported. This aligns with the positive correlation of neutrophil-mediated immunity genes positively correlated with *L. iners* detected by ref. ^41^, and the higher abundance of paucimannose, which can only be secreted by neutrophils, in CVF of women with CST III from a similar high-risk pregnant cohort^54^. Markers of activation were increased in cervical compared to peripheral neutrophils, consistent with a study of cervical lymphocytes from non-pregnant women by Prakash et al., demonstrating differential expression of CD69, CD25, HLA-DR and CCR5+ amongst blood leucocytes^55^. Furthermore, the proportion of cervical neutrophils was positively correlated with cytokine, complement proteins and matrix metalloproteinase concentrations. This is consistent with previous findings showing neutrophil counts from vaginal lavages correlate with neutrophil elastase^56^, IL-8 and IL-1β concentrations^57^, and cervical biopsies from women in-labour undergoing caesarean section showing positive correlations between neutrophil counts and IL-8, MMP-8 and MMP-9 concentrations^58^. Despite early work in murine models suggesting neutrophils were not essential for cervical remodelling preceding preterm birth^28,59^, others found neutrophil infiltration in inflammation-driven preterm models^60,61^ and was further confirmed by single-cell RNA sequencing^62,63^. It is possible that the mechanism of cervical remodelling differs across aetiologies, but also across species, and murine models are not reflective of the complexity of the human vaginal microbiome or its role in preterm birth^64^. Although we did not detect significant changes in the expression of markers of activation in cervical neutrophils in association with CST III, we detected lower CD88 (C5a receptor) and higher expression of CD63 and CD66b in association with CST IV. This may reflect activation of the effector functions, such as respiratory burst, degranulation leading to release of matrix metalloproteinases, and phagocytosis. Similar to amniotic fluid neutrophils, which are found in higher proportions in cases of intraamniotic infection and inflammation^65^ and can phagocytose less favourable bacteria such as *Streptococcus agalactiae*, *Ureaplasma urealyticum, G. vaginalis* and *E. coli*^66^, we suggest that cervical neutrophils are the main defense mechanism at the cervicovaginal interface. Despite evidence of cervical neutrophil activation, we saw minimal changes in phenotype preceding cervical shortening and spontaneous preterm birth, limiting their value as predictive markers.

In contrast, cervical shortening and sPTB were associated with higher concentrations of C1q, C4, Factor B, Factor D, C3, C3b, Factor H and Factor I, seen as early as 12–16 weeks of pregnancy. Most of our knowledge of the role of the complement system in sPTB is derived from observational studies of peripheral blood complement proteins measured at later stages of pregnancy. Higher plasma concentrations of Fragment Bb^23,24^, C3a concentrations^25,67–69^ and Factor H^70^ have been reported in women experiencing PTB. Several studies have also reported higher concentrations of amniotic fluid C3a, C4a and C5a^18,26,27^ in women with intraamniotic infection and/or delivering preterm. A paucity of studies exist reporting on CVF; Hong et al demonstrated increases in C3a and C5a from CVF taken from women in preterm labour^67^. We also demonstrated an increase in iC3b/C3b concentration in women at mid-pregnancy in advance of the development of cervical shortening and spontaneous preterm birth^15^. It is important to note the relatively high C5a concentration in CST I, as well as the increased levels of complement proteins detected in healthy individuals, but later in pregnancy. These findings indicate a plausible physiological complement-driven basal inflammation in late pregnancy in preparation for labour, but its dysregulation and early activation in the presence of sub-optimal microbiota can lead to adverse pregnancy outcomes. Our work expands on the knowledge of the contribution of the complement cascade locally at the cervicovaginal interface, and reveals the necessity of describing normal ranges in healthy individuals.

One limitation of this study is that our microbiome analysis relied on 16S rRNA gene sequencing, whereas metagenomics, metatranscriptomics and metaproteomics are emerging approaches to characterise vaginal microbiome gene content and expression, with improved strain resolution and gene pool content^71,72^. Deeper analyses of variations in strain- dependent immune responses could be of value in future studies. No single hypervariable region is sufficient to taxonomically assign all amplicon sequence variants at the species level. The choice of hypervariable region will impact taxonomic resolution and estimation of relative abundance differentially for each *Genus*^73–76^. The V1-V2 region provides sufficient information to differentiate key vaginal *Lactobacillus* species^53,77^. We have also used a mixed forward primer set^78^ to account for the poor sensitivity of the universal forward (27 F) primer to detect *Gardnerella, Bifidobacterium*, and other key vaginal taxa^79–81^. We were also limited to reporting on associations rather than direct causal mechanisms as this was an observational study conducted on human in-pregnancy samples. Direct modulation of complement function or neutrophil activation was not possible. It should also be acknowledged that the concentrations of complement proteins are lower in the female genital tract, compared to reported circulating plasma and serum concentrations^82,83^. However, we still detected statistically and clinically significant differences in adverse pregnancy outcomes. Most complement proteins reported in this study do not have ‘normal’ plasma or serum reference ranges for pregnant women, making the interpretation of ‘normal’ CVF concentrations challenging. The origin of complement proteins requires further investigation: circulating complement proteins are mainly produced by hepatocytes, and their transudation in the female genital tract may explain in part their presence in CVF. However, there is also extrahepatic complement biosynthesis from epithelial cells and immune cells, including neutrophils^84–86^, which are most likely to be responsible for local production in response to vaginal microbiota. Finally, we acknowledge the broad range of risk factors in our cohort, and were limited by numbers to perform a sub-analyses; however, the results of this study can be applied to real world setting of the most common known risk factors for sPTB.

We used the earliest timepoint collected (timepoint A, 12–14^+6^ weeks of gestation) to predict both cervical shortening and preterm birth. The model with the immune dataset alone and in combination with 16S data, outperformed the model of 16S data alone, highlighting the importance of the immune response in the aetiology of sPTB. An AUC 0.68 to predict sPTB at <37 weeks is comparable to foetal fibronectin testing (AUC 0.692) but lower than cervical length measuring (AUC 0.72)^87^. However, foetal fibronectin could only be used after 22 weeks, and its use has been withdrawn in the United Kingdom^88^; moreover, cervical length measuring requires infrastructure for surveillance and extensive training. Interpretation of these findings should be done with caution as our population is enriched with women at high risk of preterm birth, thus results cannot be extrapolated when managing a general population. Cervicovaginal metabolomic signatures can rapidly predict microbial composition and adverse outcomes^89–91^. We have previously demonstrated that metabolomic profiling of vaginal swabs using DESI-MS (desorption electrospray ionisation mass spectrometry) predicts the vaginal microbial composition and the immune response, including complement proteins (MBL, C3b/iC3b, C5, and C5a) within three minutes^90^. It is plausible that this, or similar tools, could be developed to analyse this wider panel of markers to strengthen the prediction of microbe-host immune interactions, and to perform a bedside risk stratification for cervical shortening and preterm birth. This information can be used to individualise preventative treatment, such as complement therapeutics and live biotherapeutics. The strengths of our current study include the reporting of such a broad panel of complement proteins and at early and clinically relevant timepoints, amenable to use of a predictive test in time for intervention with preventative treatments. The last decade has seen a surge in the development of complement therapeutics targeting multiple components of each pathway in phase 1–3 clinical trials, and FDA approval of anti-C3, -C5, -C1s -C5a and -C5aR1 inhibitors, including Eculizumab (anti-C5) in pregnancy^92–94^. In addition, there is evidence that Pravastatin modulates complement activity and inhibits preterm birth in LPS treated mice^95,96^, with an ongoing placebo-controlled trial to determine if Pravastatin (20 mg) prolongs gestation in women at high risk of sPTB (The PIONEER Study ISRCTN - ISRCTN93398587). Live biotherapeutics containing *L. crispatus* can lead to colonisation, as shown in a study of non-pregnant women with urinary tract infections^97^ and bacterial vaginosis (BV)^98^, with one study also reporting local pro-inflammatory cytokine suppression^99^. We have previously shown that administration of the live biotherapeutic CTV-05 (LACTIN-V), containing *L. crispatus*, is safe and well accepted by pregnant women at high risk of sPTB^100^. Although not powered to assess rates of PTB, a 50% reduction was observed compared to a historical cohort, which we hypothesise to be due to microbial and immune modulation.

In summary, our data support the contribution of the complement system in determining a dysregulated response to BV-like bacteria, and in the process leading to cervical shortening and spontaneous preterm birth. We also demonstrate that *L. crispatus* is associated with immune homoeostasis. These data support the use of complement proteins as predictive biomarkers, and the use of complement therapeutics and live biotherapeutics as novel prevention strategies for microbial-driven spontaneous preterm birth.

## Methods

### Study design

Women at high-risk of spontaneous preterm birth were recruited from preterm birth prevention clinics from four London hospitals (Chelsea and Westminster Hospital, St Mary’s Hospital, Queen Charlotte’s and Chelsea Hospital and University College London Hospital). Women were recruited and provided written informed consent, as part of the VMET 2 study (Vaginal Microbiome and Metabonome in Pregnancy, REC 14/LO/0328, approved by the NHS Health Research Authority London -Stanmore Research Ethics Committee). The study was conducted in accordance with the Declaration of Helsinki. This was not a clinical trial; therefore the Clinical Trial number is not applicable.

Inclusion criteria for recruitment included a past pregnancy experience of spontaneous preterm birth and/or preterm labour rupture of membranes, mid-trimester loss, previous fully dilated term caesarean section, cervical cerclage, history of excisional cervical treatment (cone biopsy or large loop excision of the transformation zone), or uterine anomaly. Women with an incidental finding of a short cervix (cervical length ≤25 mm measured by transvaginal ultrasound) at the time of their anomaly scan were also included in the study. Exclusion criteria included: sexual intercourse within 72 h prior to sampling, vaginal bleeding, HIV-positive women, hepatitis B-positive serology and women under 18 years of age.

Detailed metadata was collected for all women participating in the study, including maternal age, body mass index (BMI), ethnicity, blood group, gestational age at sample collection and if interventions to prevent sPTB were required (cervical cerclage and/or vaginal progesterone). BMI is measured at the time of the first appointment (between 8 and 12 weeks of gestation). Cervical length by transvaginal ultrasound was also recorded at every visit. Participant demographics are reported in Tables S1 and S2.

Matched samples of cervicovaginal fluid (CVF), peripheral blood and cytobrush were collected longitudinally when practicable across pregnancy at timepoints A (12^+0^–16^+6^ weeks of gestation), B (20^+0^–24^+6^ weeks of gestation) and C (30^+0^–34^+6^ weeks of gestation).

### Cohort description

For this study, we selected women from the VMET 2 study who had provided samples between June 2018 and June 2022. We selected all women who delivered sPTB (with samples at A + B, A, B), had a term uncomplicated pregnancy (A + B) and/or gave a cytobrush (A, B, C). Iatrogenic cases were not included. A total of 186 pregnant women were identified, providing CVF samples for metataxonomics profiling and Luminex multiplex immunoassays. A subset of women also provided blood and cytobrush samples for neutrophil immunophenotype analysis (Fig. 1A and Fig. S1A, B).

Recruited women received standard care and were followed up throughout their pregnancy. If indicated, vaginal progesterone and/or cervical cerclage were the choice of intervention as per local protocol. Further details on interventions of each cohort are available in Tables S1 and S2. Our study evaluated cervical shortening and sPTB as two separate outcomes, as both are independent, clinically relevant outcomes. The cervical shortening group included women with cervical length ≤25 mm by transvaginal ultrasound. After delivery, outcome data were retrieved. sPTB was defined as preterm delivery following either spontaneous onset of contractions (*n* = 42), PPROM (*n* = 13) or premature dilation of the cervix prior to onset of contractions (*n* = 3). Although we acknowledge there may be differences in the aetiology and pathogenesis of these phenotypes of PTB, it is highly likely that inflammation is a common pathway, since microbiome and inflammation have been widely demonstrated as key drivers of the three presentations^6–15,101,102^.

The term delivery group includes TU women, who, despite having a risk factor, delivered at term without receiving any type of intervention and TI were women who delivered at term following an intervention (cerclage and/or progesterone).

### Sample collection

CVF was sampled by trained clinicians using BBL^TM^ CultureSwab^TM^ MaxV Liquid Amies (Becton, Dickinson and Company, Oxford, UK) and Transwab (MW170 with rayon blue lid, Medical Wire & Equipment, Corsham, UK). Swabs were stored at −80 °C until use. A speculum was inserted into the vaginal canal, and the cervical os was exposed by a trained clinician. Following the collection of vaginal swabs, a cervical cytobrush (Cervical brush Q Path®, Novabrush®) was used to make ten gentle brush strokes over the anterior lip of the cervix. The brush was placed in a 50 mL conical tube containing 5 mL of PBS and passed through a sterile 40 µm cell strainer (Fisherbrand^TM^). Peripheral venous blood was collected into Lithium Heparin tubes. Peripheral venous blood was collected into Lithium Heparin tubes. Blood and cytobrush samples were analysed fresh, immediately upon collection, within 30 minutes of collection. Only cervicovaginal samples without evidence of blood contamination were used for analysis.

### DNA extraction and 16S rRNA sequencing

DNA extraction was performed as previously described^103^. Briefly, CVF collected using BBL^TM^ CultureSwab^TM^ MaxV Liquid Amies swab was thawed on ice and resuspended in Amies Liquid transport medium. Pelleted content (7000 rpm x 10 min, 4 °C) was resuspended in 300 µL lysis master mix (Lysozyme (10 mg/mL, Sigma), Mutanolysin (25000 u/mL, Sigma), Lysosthaphin (4000 U/mL) TE_50_ (10 mM TRIS/50Mm EDTA), 12% Triton, and PBS) and incubated for an hour in a 37 °C water bath. Incubation was followed by mechanical lysis using 0.1 mm glass beads (Sartorius Stedim) for 1 minute at 25 Hz in a Tissue Lyser LT (Qiagen). Finally, DNA was extracted using QiAamp DNA Mini Kit (Qiagen), following the manufacturer’s instructions.

Whole genomic DNA extracted from each swab was sequenced for the V1-V2 hypervariable regions of 16S rRNA on the Illumina MiSeq platform (Illumina, Inc. Sand Diego, California) using forward and reverse primers. The forward primer set (28f-YM) consisted of a mixture of the following primers mixed at a 4:1:1:1 ratio; 28F-Borrellia GAGTTTGATCCTGGCTTAG; 28F- Chlorlex GAATTTGATCTTGGTTCAG; 28F- Bifido GGGTTCGATTCTGGCTCAG; 28F- YM GAGTTTGATCNTGGCTCAG^78^, which enables resolution and assignment of relevant bacteria from the female reproductive tract^79–81^. The reverse primer consisted of 388 R GCTGCCTCCCGTAGGAGT. Sequencing was performed at Research and Testing Laboratories (RTL Genomics, Texas, USA).

### Luminex immunoassays

CVF collected with BBL^TM^ CultureSwab^TM^ MaxV Liquid Amies swabs was resuspended in transport liquid media and employed for measurement of cytokines (IL-1β, IL-6, IL-8, R&D Systems), complement proteins (C3a Human ProcartaPlex Simplex Kit, Invitrogen), C2, C4b, C5, C5a, Factor D, MBL, Factor I (Human Complement Magnetic Bead Panel 1, Milliplex Millipore), C3, C3b/iC3b, C4, Factor H (Human Complement Magnetic B3ead Panel 2, Milliplex Millipore) and matrix metalloproteinases (MMP-2, MMP-8, MMP-9, R&D Systems). A Transwab was resuspended in 200 µL of PBS with protease inhibitor (PI) (5 µL PI/1 mL PBS, Sigma-Aldrich) and employed to measure C1q and Factor B analytes (Human Complement Panel 2, Merck Millipore). Kits were used following the manufacturer's instructions. All samples were analysed neat except IL-8 (single-plex measurement following a tenfold dilution using Calibrator Diluent RD6-52). All samples were analysed in duplicates. Acquisition was performed with Magpix ® or Luminex 200 analysers, equipped with xPONENT^®^ software.

The sensitivity of the assay is indicated for each analyte, as per manufacturer reports: IL-1β (0.8 pg/mL), IL-6 (1.7 pg/mL) and for IL-8 (1.8 pg/mL), C1q (0.165 ng/mL), C2 (0.34 ng/mL), C4 (0.146 ng/mL), C4b (0.33 ng/mL), MBL (0.033 ng/mL), Factor B (0.022 ng/mL), Factor D (0.027 ng/mL), C3 (0.22 ng/mL), C3a (0.6 pg/mL), C3b/iC3b (4.775 ng/mL), C5 (0.8 ng/mL), C5a (0.0035 ng/mL), Factor H (0.092 ng/mL), Factor I (0.18 ng/mL), MMP-2 (108 pg/mL), MMP-8 (34.2 pg/mL), MMP-9 (13.6 pg/mL).

### Neutrophils’ characterisation by flow cytometry

Granulocytes isolated from peripheral blood following a double gradient centrifugation with Histopaque^®^-1119 and Histopaque^®^- 1077 (Sigma-Aldrich). The granulocyte layer was retrieved and washed in PBS, to be further incubated with erythrocyte lysis buffer (155 mM NH_4_Cl; 10 mM KHCO_3_; 0.1 mM EDTA) for 15 min and washed with PBS again.

Peripheral and cervical neutrophils were resuspended in PBS to 1 × 10^7^ cells/mL. A 100 µL of each suspension was pre-incubated with 2 µL of Fc Blocker (BD Biosciences) for 10 min and further stained with 7-AAD viability staining solution (BioLegend), the following anti-human antibodies (Table S7) CD45^PerCP/Cyanin 5.5^, CD15^Brilliant Violet 785^, CD63^APC^, CD66b^PE/Dazzle594^, CD11b^Alexa Fluor 700^, CD10^APC/Cyanine7^, CD33^Alexa Fluor 647^ CD35^FITC^, CD14^PerCP-eFluor710^; CD16^eFluor 450^, CD62L^BV480^, CD55^Brilliant Violet 510^, CD88^BV421^ and Brilliant Stain Buffer (BD Biosciences) for 15 min in the dark at room temperature. Markers were chosen to correctly identify neutrophils and evaluate their role in migration, adhesion, cell–cell interactions, effector functions (e.g., degranulation, respiratory burst) and interaction with the complement system^104,105^ (Table S7). After incubation, cells were washed with staining buffer (PBS, 1% BSA (Sigma), 0.1% sodium azide (Sigma)) and resuspended in 500 µL of 0.25% formaldehyde PBS prior to data acquisition with Cytek^TM^ Aurora (Cytek Biosciences). Raw spectral data were acquired and unmixed using SpectroFlo Software (Cytek Biosciences) with autofluorescence extraction. Further spectral analysis was performed with Flowjo v10 Software (Flowjo). A manual gating strategy was used to identify peripheral and cervical neutrophils (Fig. S5) to assess the MFI (Median Fluorescence Intensity) of the different activation markers.

### Sequencing processing

Microbiome analysis included the use of Cutadapt for primer sequences trimming^106^ and ASV (amplicon sequence variant) counts per sample were calculated using the QIIME2 bioinformatics pipeline (version 2022.2.1)^107^. DADA2 (version 2022.2.0)^108^ was utilised for denoising. Finally, the SILVA database^109^ was used for taxonomic classification of ASVs. The ASV count results is provided in Supplementary Information.

At the species level, samples were classified using the VALENCIA centroid classification algorithm^110^ into five community state types. Four of them were dominated by a single species of *Lactobacillus*: CST I (*L. crispatus*), CST II (L*. gasseri*), CST III (*L. iners*) and CST V (*L. jensenii*). CST IV (heterogeneous)was characterised by reduced lactobacilli and increased diversity.

### Luminex raw data examination

The primary source of raw data examination was xPONENT ^®^ software. Samples with low-bead acquisition were re-assayed. Raw data was examined for points with poor replication or outliers, which can be assessed by inspecting the %CV of replicates [%CV = STDev/Mean) * 100]; as well as the % recovery [%recovery = (observed standard value/expected standard value) * 100]. If necessary (high percentages of CV or Recovery), the standard curve could be edited by invalidating one of the standard point replicates and re-analysing). Finally, the *R*^2^ values were annotated to keep a record of the quality of fit, given data yields of *R*^2^ > 0.985. Low out-of-range concentrations were assigned half of the sensitivity of detection of the assay. Finally, the coefficient of variation was calculated across all plates (Microsoft Excel v2211) for standards and the internal pool calibrator to assess the potential plate-to-plate variation.

### Statistical analysis

Statistical analyses were performed with Graphpad Prism (v10.3.1). Categorical data were compared within groups using the chi-square or Fisher’s exact test. Comparisons of the proportion of women with different vaginal microbial compositions was also assessed with the chi-square and Fisher’s exact test. All data were tested for normal distribution and further analysed accordingly. Differences between the two groups were compared using a two-tailed Mann–Whitney *U*-test. Differences between three or more groups were calculated with the Kruskal–Wallis test, and Dunn’s post hoc multiple comparison test were used. The relative abundance of *L. crispatus* was presented as centred log-ratio (clr) transformed counts, which were computed using the propr package (v4.2.6). To evaluate associations between the relative abundance of *L. crispatus* and concentration of inflammatory mediators, Spearman’s rank correlation coefficient was calculated. To compare women with high (≥5)/low (≤5%) *L. crispatus* abundance, and high (≥median)/low <median) concentration of analytes, Chi-square and Fisher’s exact test were used. To assess complement concentrations longitudinally, women with ABC, BC and AC matched samples were selected, and a Wilcoxon matched-pairs signed rank test was used. MFI of cell surface markers was compared between matched peripheral and cervical neutrophils using the Wilcoxon matched-pairs signed rank test. For all tests, the level of statistical significance was considered as *p* value ≤0.05.

All downstream bioinformatic and statistical analysis were performed in R (R v4.0.0 was used to generate clr-transformed counts and diversity indices. All remaining bioinformatics and statistical analyses were generated using R v4.3.3). The heatmap and cluster dendrograms of the top 30 taxa by total counts and their relationship with different clinical outcomes (cervical shortening, pregnancy outcome) were generated with the ClusterHeatmap package (v2.18.0)^111^. The Shannon diversity index and richness (number of species observed) were calculated using phyloseq (v1.32.0)^112^. Linear regression modelling, Benjamini–Hochberg FDR correction for multiple testing, and calculation of the Spearman’s correlation matrix estimation were performed with R *base* functions. PCA analysis was performed with the pcaMethods (v1.94.0) package^113^. Data and analysis results were visualised using the ggplot2 (v3.5.1)^114^ and ComplexHeatmap packages. The random forest prediction models were trained and cross-validated (stratified sevenfold CV) in R (v4.5.2) using the randomForest (v4.7.1.2) and the caret (v7.0.1) packages. Missing data were imputed during the cross-validation process with k-nearest neighbour (k-5) imputation using the preProcess argument in the caret model training interface. ROC curves were plotted in ggplot2 (v3.5.2) with the plotROC package (v2.3.3).

## Supplementary information

Supplementary Information

Supplementary Information

## Data Availability

Raw sequence data have been deposited in the European Nucleotide Archive under accession codes PRJEB83084, PRJEB89979 and PRJEB90668. This study used preexisting software and did not generate new custom code or algorithms. Any additional information reported is available from the lead contact upon reasonable request.
