# Supplementary material for: Complement and neutrophils are actively involved at the cervicovaginal interface in cases of adverse microbial composition, cervical shortening and spontaneous preterm birth
Source: NPJ Biofilms Microbiomes. 2026 Apr 15;12:152. doi: 10.1038/s41522-026-00969-x (PMC13457885; doi:10.1038/s41522-026-00969-x)
Supplement: Supplementary file 1 [file 41522_2026_969_MOESM1_ESM.pdf]

Figure S1.

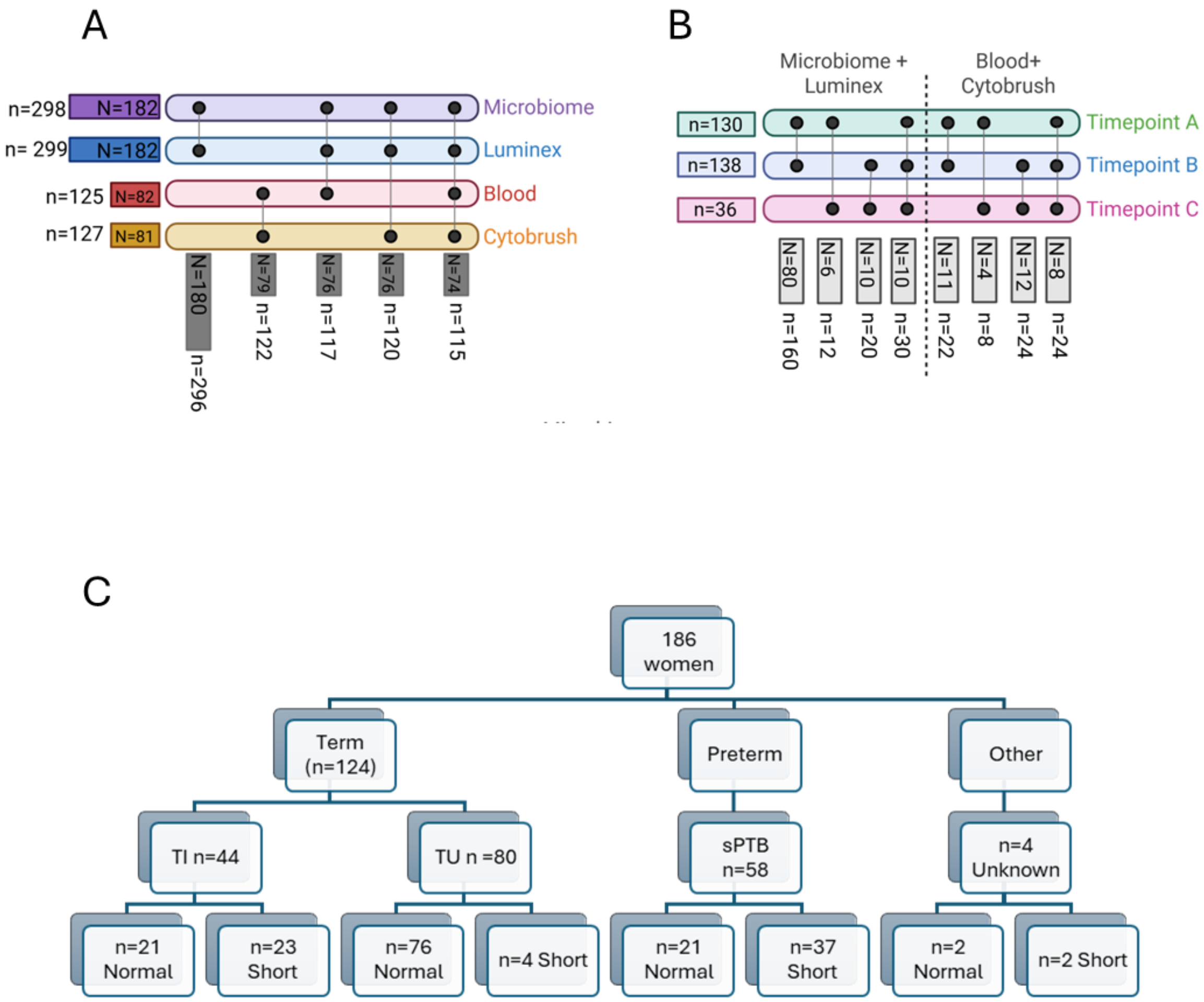

Figure S1. Sample collection, and cohort description.

A. Collection of samples (vaginal swabs, peripheral blood and cytobrush samples for 16S sequencing, Luminex immunoassays and flow cytometry) cross sectionally. N (number of women), n (number of samples). **B.** Indicates women who provided matched samples across pregnancy. Timepoint A: 12^{+0}-16^{+6}; timepoint B: 20^{+0}- 24^{+6}. Timepoint C: 30^{+0}-34^{+6}. **C.** Summary of pregnancy outcome and cervical length of study cohort. TI: term intervention (cervical cerclage and/or vaginal progesterone). TU: term uncomplicated (no interventions). sPTB (spontaneous preterm birth). Normal/short classification according to cervical length (>25mm or ≤25mm respectively) measured by transvaginal ultrasound.

Table S1. Patient demographics according to pregnancy outcome

	sPTB	TU	TI	p value
**N**	58	80	44	
sPTB 34^{+0}-36^{+6}, N (%)	36 (62%)	n/a	n/a	
sPTB <34^{+0}, N (%)	22 (38%)	n/a	n/a	
**Gestation at delivery (w+d); median**	30^{+2}	39^{+3}	38^{+5}	**** sPT vs TU; sPTB vs TI <0.0001
**Age; mean (SD)**	33 (±6)	34 (±4)	35 (±4)	* sPTB vs TU 0.0490<br>** sPTB vs TI 0.001
**BMI; median (IQR)**	25.15 (22.08-29.55)	24 (21.93-27.98)	24.5 (22.0-29.23)	0.4216
**Ethnicity, N (%)**				0.0514
White	29 (47.5%)	57 (68.8%)	22 (50%)	
Black	12 (19.7%)	7 (8.4%)	10 (22.7%)	
Other	20 (32.8%)	19 (22.9%)	12 (7.3%)	
**Blood group, N (%)**				0.1498
A	18 (31.6%)	32 (40.0%)	16 (36.4%)	
AB	1 (1.8%)	5 (6.3%)	1 (2.3%)	
B	14 (24.6%)	8 (10.0%)	4 (9.1%)	
O	24 (42.1%)	35 (43.8%)	23 (52.3%)	
**Interventions**				
**Cervical cerclage, N (%)**	31 (53.4%)	n/a	33 (75%)	
Average g. age at insertion	18^{+4}	n/a	18^{+4}	
Type of Cervical cerclage		n/a		
History	9 (29%)		14 (42%)	
USS	18 (58%)		19 (58%)	
Other	4 (13%)		0	
Suture material		n/a		
Mersilene	6 (19.4%)		0 (0%)	
Nylon	22 (71%)		31 (94%)	
Other	3 (9.7%)		2 (6%)	
**Progesterone only, N (%)**	8 (14%)	n/a	11 (25%)	n/a
**Gravida; median (IQR)**	3 (2-4)	2 (1-3)	3 (2-4)	* sPTB vs TU 0.0341<br>*TU vs TI 0.0136
**Parity; median (IQR)**	1 (0-2)	0 (0-1)	1 (0-1)	0.0868
**Index Risk factor, N (%)**				**** <0.0001
Uterine anomaly	1	5	2	
Uterine anomaly+other	1	0	0	
Cx treatment	3	38	8	
Cx treatment + other	2	0	4	
sPTB	8	3	0	
sPTB+MTL	2	0	2	
sPTB+PPROM	11	10	4	
sPTB+other	5	6	6	
MTL	6	4	0	
MTL+PPROM	3	8	3	
MTL+other	3	2	5	
PPROM	1	0	0	
Prev cerclage	0	0	2	
Incid. finding short cervix	11	0	7	
Other	1	0	0	
FDCS	0	4	1	

Abbreviations, sPTB: spontaneous preterm birth. TU: term uncomplicated. TI: term intervention. SD: standard deviation. Gestation at delivery (w: weeks; d: days). IQR: interquartile range. BMI: body mass index. Average g. age: Average gestational age. USS: ultrasound indicated. PPROM: preterm prelabour rupture of membranes. MTL: mid trimester loss. Risk factor + other indicates multiple risk factors (main risk factor plus any other of the listed ones). Other: medical TOP with chorioamnionitis. FDCS: fully dilated caesarean section at term. Ethnicity other: Asian and mixed race.

Differences between the three groups were assessed with Kruskal-Wallis with Dunn's multiple comparisons test, except for the variable "age" which was assessed with one-way ANOVA. The proportions of "Ethnicity" and "ABO" variables were assessed with Chi-square. P value summary: * $p < 0.05$; ** $p < 0.01$, **** $p<0.0001$

Table S2. Patient demographics according to cervical length

	Short cervix	Long cervix	p value
**N**	66	120	
**Pregnancy outcome**			****<0.0001
sPTB, N (%)	37 (57.8%)	21 (17.8%)	
TU, N (%)	23 (35.9%)	76 (64.4%)	
TI, N (%)	4 (6.3%)	21 (17.8%)	
**Shortest cervical length (mm); median (IQR)**	16.5 (9.8-21)	31 (30-34)	**** <0.0001
**G. age at shortening (w+d); median**	21^{+0}	n/a	
**Latency to delivery (days); median (IQR)**	76.5 (23-122)	n/a	
**Age; median (IQR)**	33 (30-36.25)	34 (31-37)	0.1678
**BMI; median (IQR)**	26 (22.1-30)	24 (22-28)	0.0925
**Ethnicity, N (%)**			*0.0112
White	30 (45.5%)	78 (65%)	
Black	16 (24.2%)	12 (10%)	
Other	20 (30.3%)	30 (25%)	
**Interventions**			
**Cervical cerclage**	49 (74.2%)	16 (13.3%)	
G. age at insertion (w+d); median	20^{+5}	15^{+5}	
Type of Cervical cerclage			
History	8	15	
USS	37	1	
Other	4	0	
Suture material			
Mersilene	9	0	
Nylon	37	16	
Other	3	0	
**Progesterone only, N (%)**	7 (10.6%)		
**Gravida; median (IQR)**	3 (1.8-4)	2 (2-3)	0.194
**Parity; median (IQR)**	1 (0-1)	0 (0-1)	0.4588

Abbreviations, sPTB: spontaneous preterm birth. TU: term uncomplicated. TI: term intervention. mm: milimiters. IQR: interquartile range. Gestational age (w+d): weeks + days. BMI: body mass index. USS: ultrasound indicated. Ethnicity other: Asian and mixed race.

Differences between two groups were assessed with Mann-Whitney test. Ethnicity proportions were assessed with Chi-square. P value summary: * $p < 0.05$; **** $p < 0.0001$

Figure S2.

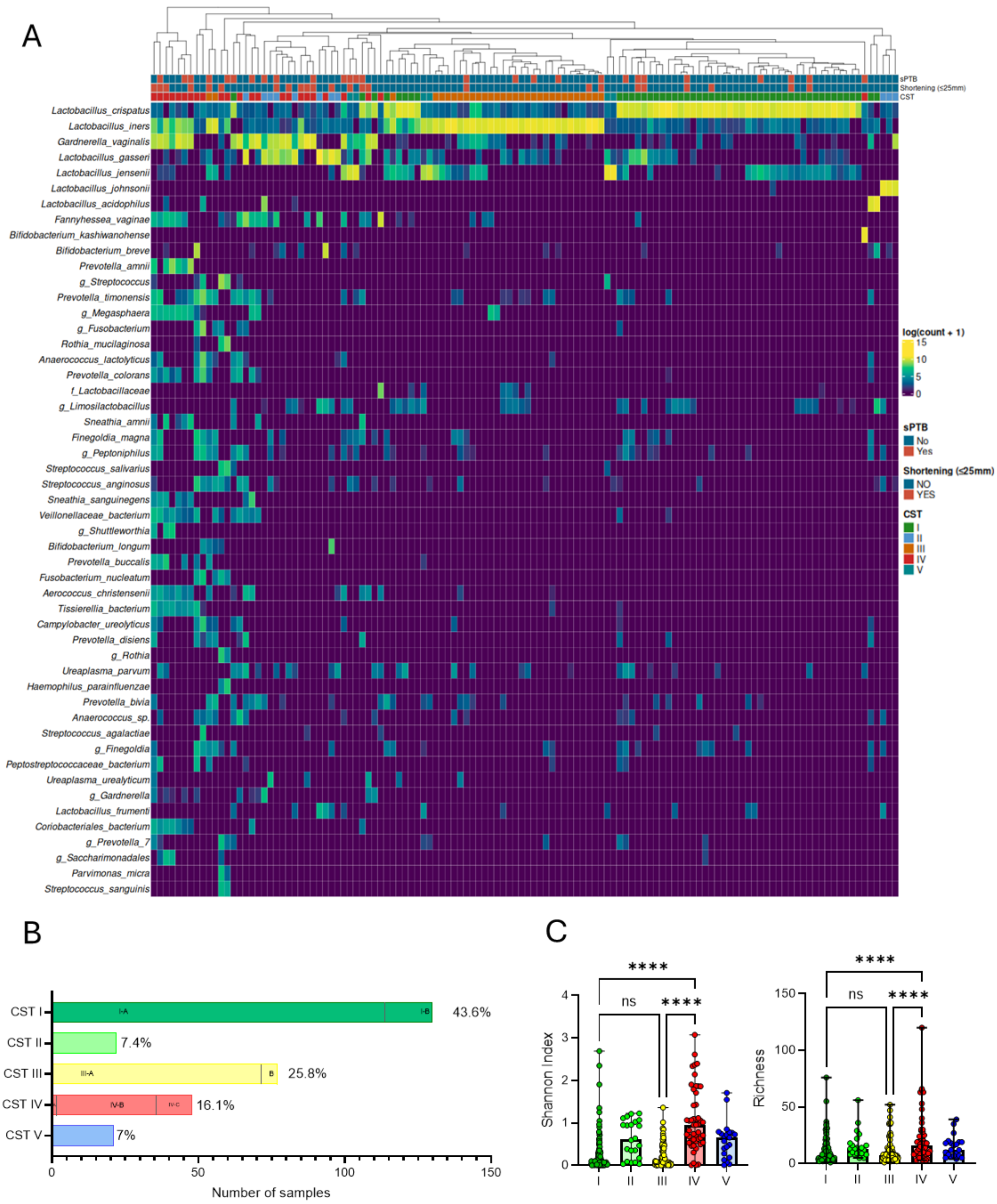

D

	Diversity	Normal	Short	Mann-Whitney
Timepoint A	Shannon	0.09037 (0.03051-0.4845)	0.357 (0.04558-1.040)	0.0977
	Richness	7.5 (5-13)	9 (5-19.5)	0.3198
Timepoint B	Shannon	0.1206 (0.03519-0.5428)	0.4999 (0.05403-0.9329)	* 0.0276
	Richness	7 (5-14)	9.5 (5-22)	0.2774
	Diversity	TU	sPTB	Mann-Whitney
Timepoint A	Shannon	0.09683 (0.03212-0.5825)	0.3525 (0.03051-1.048)	0.0943
	Richness	7 (5-120	11 (5-27)	0.0686
Timepoint B	Shannon	0.1017 (0.03158-0.4809)	0.3784 (0.05237-0.9642)	* 0.0127
	Richness	7 (5-13)	8 (5-22)	0.2412

Figure S2. Vaginal microbial composition of study cohort

A. Heatmap shows distribution of samples in top 50 species level taxa, according to pregnancy outcome, cervical length and by Community State Type (CST). Raw counts were log transformed. Hierarchical clustering analysis and dendrogram of samples (columns) was obtained using Euclidean distance and complete linkage. **B.** Number of samples classified as Community State Type - CST I (total, I-A, I-B), CST II, CST III (total, III-A, III-B), CST IV (total, IV-A, IV-B, IV-C) and CST V. Comparisons were made using Kruskal-Wallis with Dunn's multiple comparisons test. **C.** Shannon Index and Richness (number of species observed) according to CST. **D.** Diversity indexes according to cervical length and pregnancy outcome. Comparisons were made with one-tailed Mann-Whitney. P value summary: * $p < 0.05$; ** $p < 0.01$; **** $p < 0.0001$

Table S3. CST Data

	Timepoint A						
**Analyte**	**CST I**	**CST II**	**CST III**	**CST IV**	**CST V**	**KW**	**p.adj**
IL-1β	24.07 (7.481-98.73)	72.3 (32.68-200.5)	96.11 (27.31-298.4)	70.56 (25.68-762.4)	202.1 (6.698-377.5)	** 0.0036	0.0326
IL-6	0.85 (0.4434-2.461)	0.85 (0.3249-1.305)	0.85 (0.6900-3.898)	1.332 (0.8500-10.33)	1.656 (0.7712-35.86)	* 0.0453	0.1386
IL-8	656 (274.1-2168)	1216 (602.1-3696)	1562 (633.7-4535)	706 (105.5-3027)	861 (381.0-5427)	0.0561	0.1919
C1q	94.78 (55.76-211.9)	495.8 (251.1-1144)	147.6 (87.72-298.5)	2067 (705.2-4423)	150.3 (71.25-546.4)	****<0.0001	****0.00001
MBL	250.3 (82.72-516.7)	186.8 (89.22-633.7)	245.7 (134.8-775.9)	171.4 (74.39-733.4)	127.2 (24.52-294.9)	0.8975	0.7549
Factor B	24.88 (11.00-110.0)	170.7 (45.07-1372)	42.7 (11.00-139.6)	301.8 (80.85-3210)	46.67 (11.00-1671)	***0.0003	**0.0076
Factor D	13.5 (13.50-62.22)	27.98 (13.50-442.90)	51.36 (13.50-123.2)	185.1 (37.55-551.2)	65.71 (20.27-1848)	*** 0.0002	**0.0045
C2	10343 (3928-20650)	6643 (3361-17223)	8913 (3599-15387)	5337 (1386-12622)	4681 (4512-10409)	0.1742	0.3932
C4	4203 (912.3-13071)	24273 (4785-47083)	22625 (5405-42436)	63751 (9407-184370)	22987 (4317-69802)	****<0.0001	**0.0092
C4b	547.9 (165.0-885.8)	508.7 (312.2-1069)	322.1 (165.0-896.4)	1218 (556.6-4101)	165 (165.0-1604)	** 0.0029	*0.0132
C3	110 (110.0-110.0)	110 (110.0-732.0)	110 (110.0-112.7)	825.3 (110.0-2582)	110 (109.0 -1372)	** 0.0014	0.0519
C3a	313.2 (240.5-612.9)	387.6 (293.0-640.7)	432.7 (286.2-863.1)	237 (21.94-485.6)	280.6 (105.9-578.0)	** 0.0056	*0.0309
C3b	3132 (2388-4795)	8257 (2388-47736)	5594 (2487-35225)	101847 (15935-245399)	7529 (3963-406275)	****<0.0001	***0.0007
C5	875.2 (400.0-1440)	400 (400.0-1203)	925.8 (400.0-2147)	650.3 (400.0-2484)	604.5 (400.0-1004)	0.9967	0.8530
C5a	22.31 (7.668-44.37)	7.257 (4.151-20.82)	17.41 (6.757-27.96)	3.637 (0.1170-21.04)	958 (0.001750-16.91)	** 0.0052	*0.0114
Factor H	10090 (4426-31949)	18370 (7453-44073)	29272 (11600-64361)	48297 (11541-78163)	29531 (5512-62565)	** 0.0024	*0.0299
Factor I	90 (90.00-267.9)	552.5 (90.00-2773)	121.5 (90.00-1129)	1864 (557.1-4891)	90 (90.00-7947)	****<0.0001	*0.00001

	Timepoint B						
**Analyte**	**CST I**	**CST II**	**CST III**	**CST IV**	**CST V**	**KW**	**p.adj**
IL-1β	29.96 (9.196-102.9)	108.1 (52.25-248.9)	118.2 (40.77-276.9)	508 (93.58-1006)	36.42 (21.63-164.1)	**** <0.0001	1.09E-04
IL-6	0.85 (0.8500-1.816)	0.85 (0.8226-4.521)	0.85 (0.5278-1.882)	0.85 (0.4062-4.275)	0.274 (0.1920-0.8500)	0.8915	0.276533333
IL-8	680.4 (315.5-2239)	1700 (625.0-2464)	1800 (701.4-3372)	1800 (484.9-6859)	865 (552.7-1866)	* 0.0132	0.06222
C1q	119.1 (51.08-364.7)	1008 (343.6-3225)	241.2 (64.54-927.7)	2428 (822.8-6818)	297.8 (191.0-1058)	**** <0.0001	**0.0067
MBL	167.1 (57.66-582.2)	515.7 (287.6-2436)	309.2 (139.3-900.6)	511.9 (178.9-841.9)	234.9 (36.02-373.0)	0.1021	0.0835
Factor B	58.41 (11.00-110.0)	111.4 (38.17-1697)	67.88 (11.00-800.9)	259.8 (109.9-3265)	195.1 (11.00-343.2)	*** 0.0003	0.0512
Factor D	32.6 (13.50-153.6)	109 (13.50-231.9)	67.16 (13.84-133.1)	305.7 (43.85-591.2)	60.05 (13.50-369.6)	** 0.0071	0.0512
C2	9562 (3523-21011)	13898 (5387-19569)	9281 (4661-18571)	8964 (3597-18658)	7551 (4813-18120)	0.8853	0.966
C4	7000 (1272-52270)	35592 (15559-144990)	23022 (7213-66139)	151707 (14357-208715)	13557 (5805-60950)	*** 0.0001	0.0641
C4b	477.6 (165.0-1299)	1528 (861.2-3425)	489.6 (173.7-1119)	2050 (927.3-5506)	620.3 (312.2-956.6)	** 0.0041	**0.0067
C3	110 (110.0-118.0)	110 (78.81-110.0)	110 (110.0-167.8)	365.1 (110.0-1114)	110 (110.0-136.9)	** 0.0018	0.0882
C3a	473.6 (226.6-880.3)	516.9 (194.9-902.8)	520.6 (265.9-987.8)	457.2 (115.2-733.5)	365.8 (238.7-610.6)	0.5317	0.7797
C3b	4575 (2388-21900)	36803 (16623-57926)	13354 (2568-29789)	70842 (32422-307480)	7371 (3616-15937)	**** <0.0001	0.0529
C5	400 (400.0-1299)	1299 (400.0-5299)	926.8 (502.3-2721)	824.7 (400.0-2717)	663.9 (400.0-2721)	0.0723	0.12981
C5a	21.54 (6.469-47.05)	22.73 (16.89-35.64)	22.2 (10.92-34.89)	11.15 (2.610-24.97)	9.609 (4.206-21.16)	0.165	0.2368
Factor H	19181 (6659-53736)	33626 (23791-111386)	39564 (13448-67899)	57637 (36609-96174)	18116 (9218-21391)	** 0.0066	0.0628
Factor I	90 (90-1717)	1519 (1223-1758)	614.5 (90-1737)	4021 (864.5-7621)	842.3 (90-950.7)	*** 0.0001	**0.002

Table S3. Cervicovaginal fluid analytes concentration according to CST microbial composition. Timepoint A: 12^{+0}-16^{+6} weeks of gestation. Timepoint B: 20^{+0}-24+6 weeks of gestation. Differences were assessed with Kruskal Wallis with Dunn's multiple comparisons test. P value summary: * p < 0.05; ** p < 0.01; *** p <0.001; **** p < 0.0001

Table S4 Correlations between percentage *L. crispatus* and concentration of immune mediators according to cervical length and pregnancy outcome

	CERVICAL SHORTENING						PREGNANCY OUTCOME					
	Timepoint A			Timepoint B			Timepoint A			Timepoint B		
**Analyte**	**Spearman r**	**p value**	**Chi-square**	**Spearman r**	**p value**	**Chi square**	**Spearman r**	**p value**	**Chi square**	**Spearman r**	**p value**	**Chi square**
IL-1β	-0.335	*** 0.0001	0.153	-0.417	**** <0.0001	0.4791	-0.2905	** 0.0031	** 0.0044	-0.3738	**** <0.0001	* 0.0488
IL-6	-0.1158	0.2023	0.3288	0.04705	0.5879	0.6898	-0.01591	0.8745	0.6304	0.06949	0.4666	0.6109
IL-8	-0.1224	0.174	0.49	-0.1786	* 0.0397	0.1019	-0.08764	0.3787	* 0.0481	-0.1313	0.1716	0.1776
C1q	-0.4285	**** <0.0001	* 0.0141	-0.3724	**** <0.0001	0.4227	-0.4416	**** <0.0001	* 0.0129	-0.3053	** 0.0012	* 0.0464
MBL	0.02908	0.7455	0.4447	-0.1603	0.0643	0.0569	0.003872	0.9687	0.4717	-0.09327	0.328	* 0.0284
Factor B	-0.2383	** 0.0085	* 0.0126	-0.3188	*** 0.0002	0.9018	-0.2155	* 0.0296	0.3046	-0.3225	*** 0.0007	0.2542
Factor D	-0.3459	**** <0.0001	0.2708	-0.2822	*** 0.0010	0.4333	-0.2674	** 0.0058	0.39	-0.2951	** 0.0016	* 0.0490
C2	0.1294	0.1472	0.3676	0.05767	0.5113	0.165	0.1473	0.1337	0.7278	0.1125	0.2418	0.0963
C4	-0.4356	**** <0.0001	* 0.0201	-0.367	****<0.0001	0.2622	-0.3712	**** <0.0001	** 0.0075	-0.3372	*** 0.0003	* 0.0460
C4b	-0.09299	0.2984	0.3591	-0.2586	** 0.0026	0.7343	-0.02891	0.7697	0.4717	-0.1702	0.0729	0.5237
C3	-0.145	0.1039	0.508	-0.2172	* 0.0117	0.7286	-0.131	0.1828	0.0562	-0.2382	* 0.0114	* 0.0121
C3a	0.003069	0.9728	0.2223	-0.07302	0.4018	0.0882	0.06649	0.5025	0.2436	-0.02664	0.7814	* 0.0392
C3b	-0.4551	****<0.0001	* 0.0253	-0.3282	*** 0.0001	0.1323	-0.4245	**** <0.0001	* 0.0182	-0.3233	*** 0.0005	**0.0091
C5	-0.01834	0.8378	0.4238	-0.2309	** 0.0073	0.2955	-0.02228	0.8215	** 0.0067	-0.2798	** 0.0028	0.4018
C5a	0.2151	* 0.0152	0.3405	0.06037	0.4883	0.8229	0.2257	* 0.0206	0.7697	0.1066	0.2633	0.3088
Factor H	-0.2625	** 0.0029	0.1783	-0.2017	* 0.0199	* 0.0298	-0.2336	* 0.0165	0.0621	-0.2148	* 0.0236	0.0765
Factor I	-0.3838	****<0.0001	** 0.0091	-0.3564	****<0.0001	* 0.0118	-0.322	*** 0.0008	**** <0.0001	-0.3751	**** <0.0001	** 0.0031

Table S4. Associations between *L. crispatus* abundance, concentration of immune mediators and outcomes. Timepoint A: 12^{+0}-16^{+6} weeks of gestation. Timepoint B: 20^{+0}-24^{+6} weeks of gestation. Correlations between clr *L. crispatus* counts and concentration of immune mediators were assessed with Spearman correlation. Proportions of women in high-risk/low-risk quadrants evaluated with Chi-square. P value summary: * p < 0.05; ** p < 0.01; *** p <0.001; **** p < 0.0001

Figure S3.

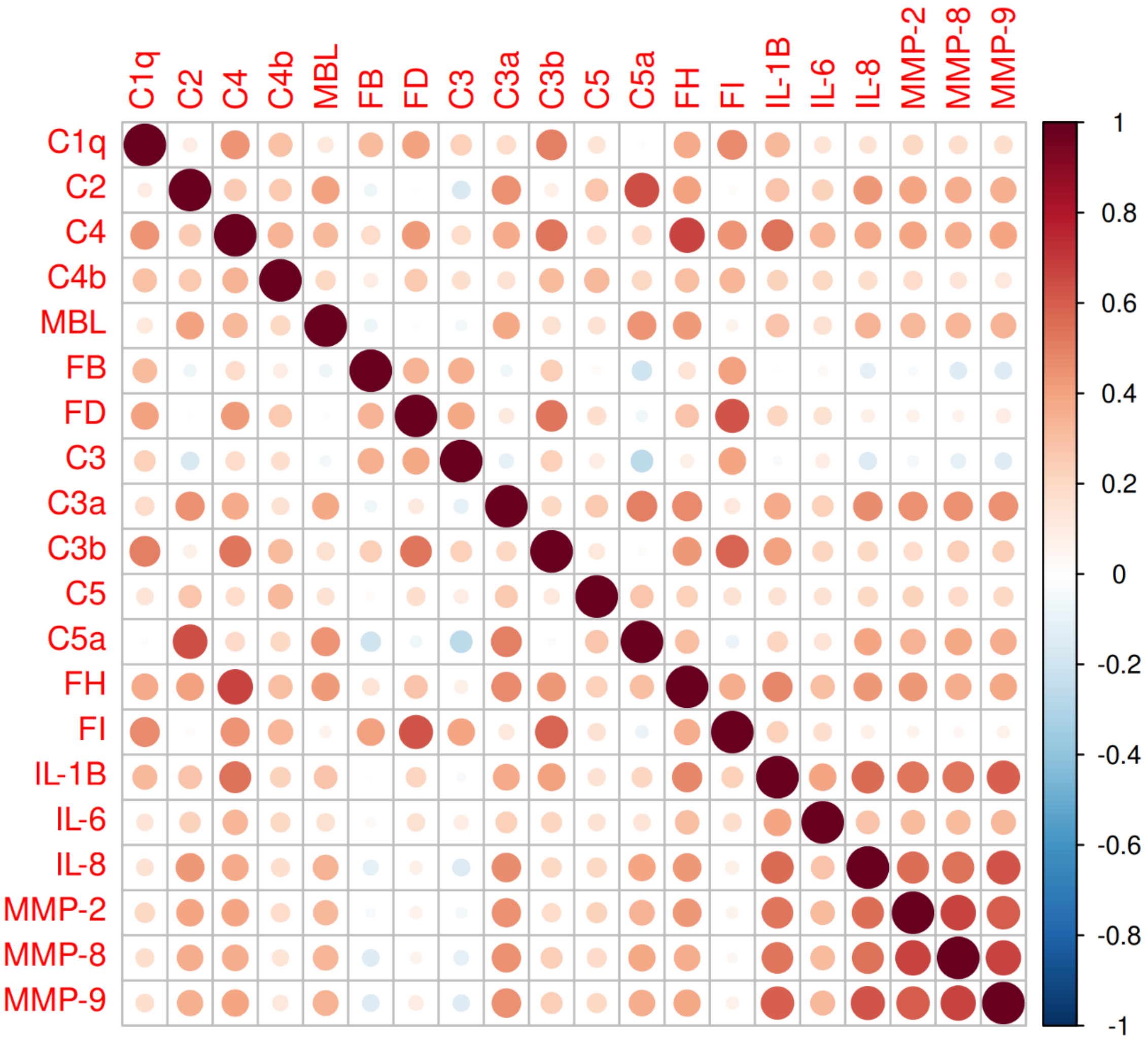

Figure S3. Correlation matrix of immune mediators. Correlation matrix between concentration of cytokines, complement proteins and matrix metalloproteinases. Colour indicates positive (blue) or negative (red) correlation. Dot size indicates correlation strength.

Figure S4

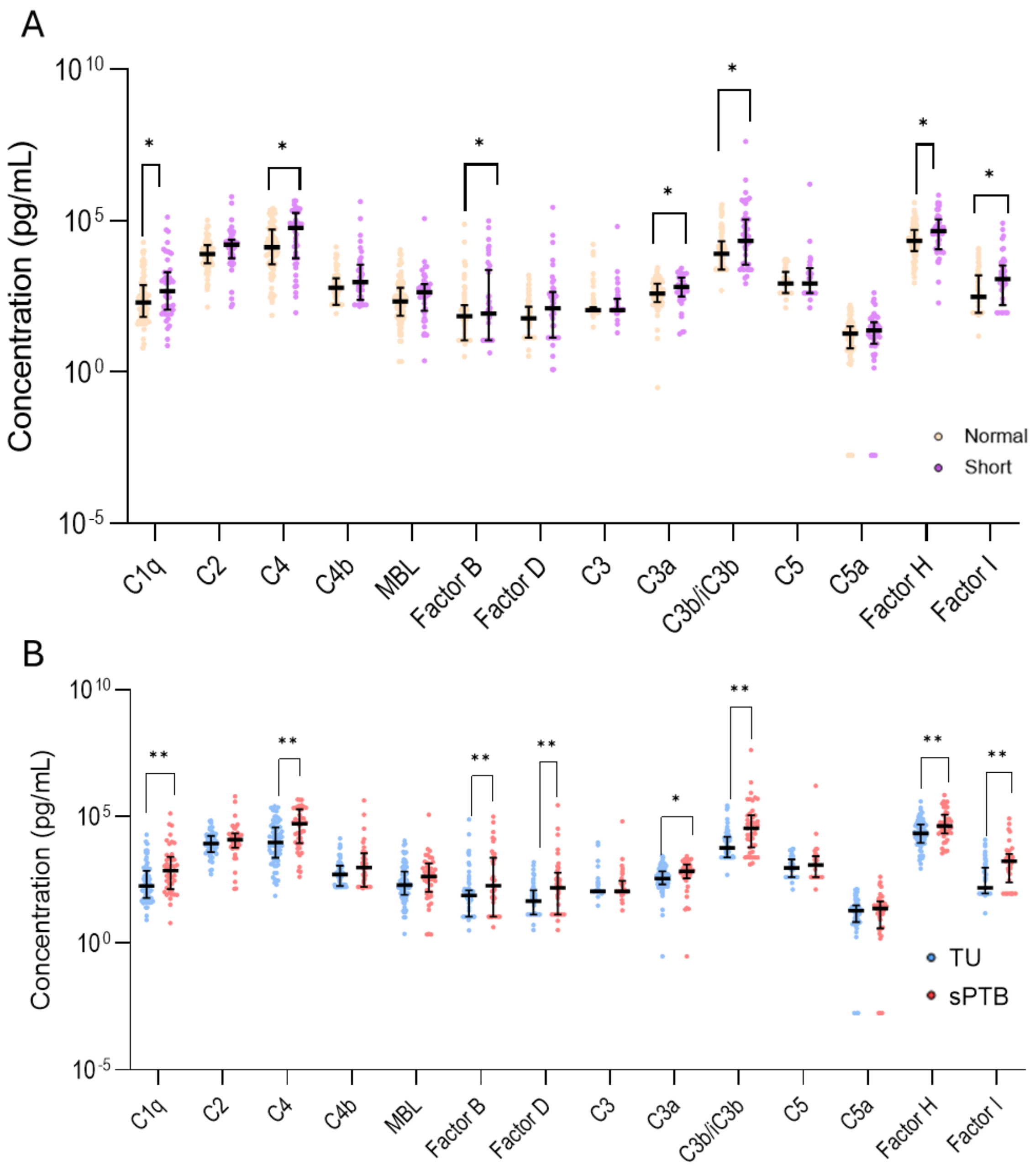

Figure S4. Cervicovaginal fluid complement protein concentrations at timepoint B. Timepoint B: 20^{+0}-24^{+6} weeks of gestation. A) According to cervical shortening. B) According to pregnancy outcome. Median and interquartile ranges of C1q, C2, C4, C4b, MBL, Factor B, Factor D, C3, C3a, C3b/iC3b, C5, C5a, Factor H and Factor I (all pg/mL). Differences were assessed with two-tailed Mann-Whitney test. P value summary: * $p < 0.05$; ** $p < 0.01$; *** $p < 0.001$; **** $p < 0.0001$

Table S5. Complement concentration by cervical length and pregnancy outcome statistical analyses

	CERVICAL SHORTENING							
	Timepoint A				Timepoint B			
Analyte	Normal	Short	P value	BH	Normal	Short	P value	BH
C1q	140.1 (81.91-531.0)	576 (189.4-4423)	***0.0005	*0.0452	198.7 (65.97-732.1)	463.8 (114.1-1955)	*0.0139	*0.01638
MBL	204.8 (80.86-405.8)	249.6 (97.96-1198)	0.1873	0.238	214 (72.18-607.1)	430.8 (105.8-798.9)	0.0908	0.1375
Factor B	43.89 (11.00-134.9)	177.8 (71.47-1260)	**0.0021	*0.0452	69.09 (11.00-159.2)	85.21 (11.00-2355)	0.3673	*0.01638
Factor D	27.98 (13.50-115.7)	113 (25.73-185.5)	*0.0179	*0.0452	58.07 (13.50-142.1)	128.3 (13.50-438.9)	*0.0334	0.0734
C2	7792 (3422-14334)	9354 (3521-23533)	0.2804	0.3005	7771 (3884-15750)	15897 (5758-23704)	* 0.0209	0.05565
C4	7918 (2165-36012)	36683 (10356-130149)	*** 0.0007	**0.0075	12971 (3560-50207)	56894 (5632-178513)	** 0.0081	*0.01829
C4b	477.6 (165.0-929.0)	941.2 (165.0-3003)	0.0753	0.1325	609.4 (165.0-1240)	929 (237.6-3449)	0.0722	0.12964
C3	110 (110.0-120.9)	115.4 (110.0-1260)	* 0.0431	0.175	110 (110.0-139.0)	115.4 (110.0-263.5)	0.4975	0.577
C3a	319.2 (195.6-508.0)	567.6 (242.7-917.1)	0.0917	0.1425	391.5 (205.2-822.8)	639.3 (309.5-1323)	*0.0193	*0.0498
C3b	4262 (2388-16545)	25734 (4735-175492)	** 0.0022	*0.0452	8155 (2388-20897)	21256 (3493-110451)	** 0.0090	*'0.01829
C5	809.1 (400.0-1299)	925.8 (400.0-3331)	0.2577	0.2998	824.7(400.0-2005)	824.7 (400.0-2724)	0.4833	0.577
C5a	15.08 (4.004-26.66)	17.19 (4.414-45.76)	0.3208	0.32	18.45 (6.000-32.47)	23.57 (8.550-43.95)	0.233	0.3442
Factor H	15432 (5440-46844)	39006 (13102-145217)	** 0.0018	**0.0039	21664 (9826-48862)	45449 (11436-111875)	**0.0080	*0.011998
Factor I	90 (90.00-882.2)	879.7 (105.7-3020)	**0.0022	*0.0117	304.9 (90.00-1546)	1187 (162.9-3320)	**0.0020	*0.01638

	PREGNANCY OUTCOME							
	Timepoint A				Timepoint B			
Analyte	TU	sPTB	P value	BH	TU	sPTB	P value	BH
C1q	132.7 (66.47-479.5)	356.5 (122.6-2118)	**0.0071	*0.04935	181.3 (60.77-709.4)	715.8 (136.7-2563)	**0.0011	**0.006
MBL	212.7 (67.38-374.3)	253.5 (105.8-1742)	0.1015	0.204	195.1 (81.60-661.6)	430.8 (105.8-1411)	0.0793	0.1238
Factor B	50.63 (11.00-123.5)	119.4 (11.00-1260)	0.0578	0.1666	77 (11.00-119.4)	188.3 (11.00-2355)	*0.0319	**0.0064
Factor D	27.65 (13.50-93.00)	44.21 (13.50-237.7)	0.1378	0.2415	44.78 (13.50-121.3)	153.6 (13.50-597.5)	**0.0045	**0.00966
C2	7686 (3246-13593)	8338 (4072-26971)	0.1656	0.2567	8560 (3889-17009)	12089 (5899-21156)	0.1772	0.2253
C4	7780 (1893-33126)	30496 (9962-123955)	***0.0003	**0.001974	9364 (2354-37340)	50207 (8764-189353)	***0.0008	**0.0011
C4b	473.2 (165.0-952.2)	643.1 (165.0-2514)	0.3077	0.4298	513.9 (182.4-1153)	968.9 (165.0-3449)	0.1408	0.1974
C3	110 (110.0-112.7)	110 (110.0-359.0)	0.4915	0.5273	110 (110.0-118.0)	110 (110.0-287.3)	0.5798	0.5783
C3a	312.9 (175.4-519.5)	387.6 (148.5-946.8)	0.543	0.5826	354.2 (208.0-675.4)	677.1 (364.6-1267)	*0.0144	*0.0263
C3b	3922 (2388-10862)	8257 (3029-177049)	*0.0127	*0.02254	5659 (2388-15746)	33559 (6062-110451)	***0.0002	**0.0011
C5	816.9 (400.0-1808)	491.5 (400.0-1958)	0.9553	0.957	925.8 (400.0-2005)	1203 (400.0-2724)	0.1977	0.2298
C5a	16.75 (3.446-27.71)	15.08 (4.729-49.19)	0.435	0.5273	19.25 (6.728-31.49)	23.57 (3.786-43.95)	0.5845	0.584
Factor H	13360 (5147-47005)	44045 (13128-79927)	**0.0079	*0.04935	21629 (9081-47446)	41906 (21329-117439)	**0.0054	**0.006
Factor I	90 (90.00-700.6)	278.5 (90.00-3232)	0.071	0.1666	152.9 (90.00-970.5)	1704 (251.2-3320)	****<0.0001	**0.0011

Table S5. Statistical analysis of complement protein concentration by cervical length and pregnancy outcome. Timepoint A: 12^{+0}-16^{+6} weeks of gestation. Timepoint B: 20^{+0}-24^{+6} weeks of gestation. Differences were assessed with two-tailed Mann-Whiteny test. P value summary: * $p < 0.05$; ** $p < 0.01$; *** $p < 0.001$

Figure S5

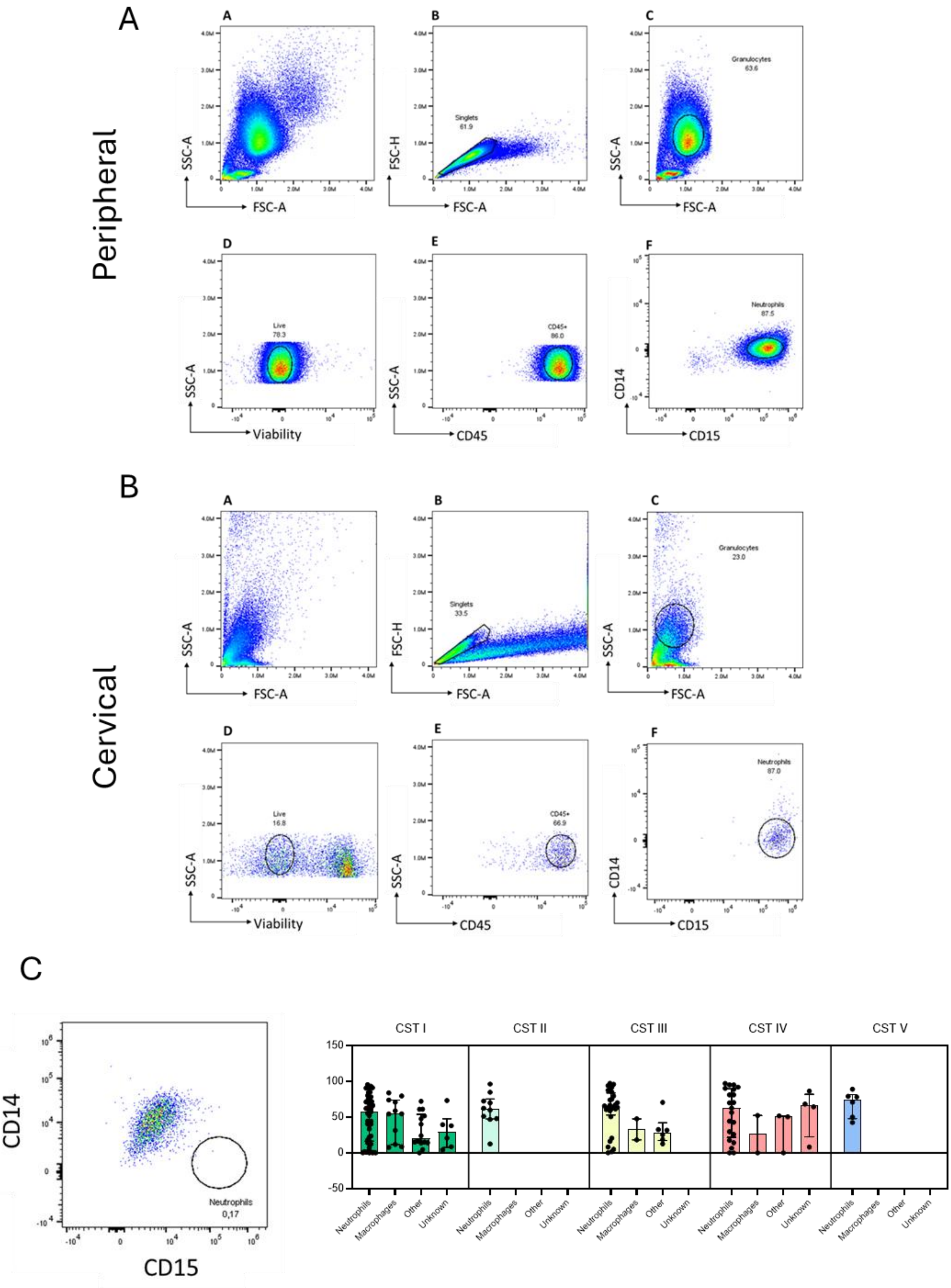

Figure S5. Flow cytometry gating strategy. A. Peripheral neutrophils. **B.** Cytobrush samples. In all cases, cells are visualised on the FSC-A, SSC-A. Cell doublets were excluded by setting gates in the FSC-A (area) vs FSC-H (height). Granulocyte population was detected on the FSC-A, SSC-A. Live cells were

gated on the viability dye-negative population. Immune cells are selected in the $CD45^+$ gate. Neutrophils are detected in the $CD15^+$ $CD14^{-/low}$ population. **C.** Identification of macrophages as $CD45^+$, $CD15^-$, $CD14^+$; and identification of the proportion of cervical immune cells according to microbial composition.

Table S6.

A) Peripheral vs cervical

Marker	Peripheral neutrophils	Cervical neutrophils	Wilcoxon test	P. adj
CD10	21325 (16719-25961)	17411 (12505-30989)	0.9668	0.966616
CD11b	17994 (13582-26147)	7255 (3881-13103)	**** <0.0001	1.08E-06
CD16	131176 (89091-224702)	21510 (13693-33941)	**** <0.0001	5.23E-11
CD33	4384 (2998-6274)	3684 (2850-4973)	* 0.0361	0.045828
CD35	22298 (18898-27217)	4604 (3169-15330)	**** <0.0001	4.48E-10
CD55	65780 (53206-78007)	65953 (52940-82797)	0.7018	0.77754
CD62l	25533 (16408-35704)	2219 (1746-3902)	**** <0.0001	2.79E-10
CD63	7789 (5107-10461)	14539 (8708-45720)	**** <0.0001	1.33E-08
CD66b	15405 (12607-20414)	14759 (5774-59243)	*0.0317	0.045828
CD88	4384 (3236-5923)	20329 (6402-41361)	**** <0.0001	3.39E-10

B) MFI By Microbial composition

	Marker	CST I	CST II	CST III	CST IV	CST V	Kruskal Wallis	P. adj
Peripheral neutrophils	CD10	21242 (16738-25674)	19747 (14841-28801)	23094 (19141-27978)	21333 (16978-26120)	19390 (13792-22778)	0.6201	0.6867
	CD11b	18371 (13482-26376)	13861 (7540-24879)	18766 (16383-27824)	20786 (13404-31426)	15817 (13236-19226)	0.7782	0.6867
	CD16	130486 (77945-227509)	136604 (86617-220691)	137195 (92480-224252)	140097 (97356-235965)	104372 (60951-193830)	0.7008	0.6867
	CD33	4422 (2652-6661)	4331 (3431-7825)	4347 (3164-5604)	4497 (3363-5568)	3895 (2317-6538)	0.9598	0.9160
	CD35	22279 (18473-27765)	22844 (18851-33089)	20738 (17824-24626)	26616 (22221-28646)	20424 (16739-23626)	*0.0436	0.4033
	CD55	68833 (59957-78156)	64308 (50972-74651)	60637 (50096-66866)	59659 (54027-81214)	76916 (55693-91102)	0.0863	0.4033
	CD62L	23826 (16190-35987)	16840 (12515-33952)	27774 (18346-38660)	25392 (18325-34108)	32149 (15189-44871)	0.5609	0.6867
	CD63	7365 (5766-10772)	7689 (3895-12035)	7806 (2840-10250)	7954 (4376-10179)	8037 (6142-12192)	0.6244	0.6867
	CD66b	15048 (12186-20838)	17839 (14260-26447)	13964 (10015-18338)	18948 (13378-21657)	15405 (13300-19369)	0.1171	0.4033
	CD88	4424 (3414-6292)	3506 (2410-5002)	3848 (3007-5131)	4625 (2597-6118)	5033 (3914-6539)	0.3612	0.6867
Cervical neutrophils	CD10	26463 (14013-58933)	13496 (8359-18699)	13991 (12073-17310)	17119 (11796-22220)	13356 (8232-18479)	* 0.0158	0.0525
	CD11b	6345 (3871-9720)	9163 (4344-19089)	7651 (2663-26286)	8225 (3869-22039)	3080 (2157-4002)	0.5046	0.6522
	CD16	23006 (14233-31023)	18720 (12345-45443)	28960 (14310-44073)	17258 (7946-30760)	4723 (3514-5932)	0.205	0.4000
	CD33	3633 (2660-4670)	3903 (3447-5723)	3380 (2841-5070)	4104 (3321-6114)	2266 (1468-3064)	0.6684	0.6522
	CD35	4217 (3151-14931)	9998 (2557-22351)	6427 (3021-15497)	7573 (3182-16768)	2830 (2537-3123)	0.674	0.6522
	CD55	70873 (52490-80623)	61013 (37665-157507)	64713 (59365-71240)	87402 (54313-155498)	53655 (52069-55241)	0.3861	0.6522
	CD62L	2153 (1531-3736)	3478 (2230-6462)	2690 (1750-7344)	2138 (1625-3511)	1859 (1685-2032)	0.5948	0.6910
	CD63	11987 (8268-24010)	25934 (9507-126883)	14525 (9201-20848)	70312 (14927-173954)	13254 (8066-18441)	**0.0047	*0.0455
	CD66b	8752 (4944-25920)	72588 (28933-86576)	12951 (8309-108509)	48295 (16505-113973)	6137 (5699-6574)	*0.0183	0.0525
	CD88	36394 (16994-56615)	8349 (3861-18556)	17638 (4937-25864)	15642 (4792-22262)	9618 (6186-13049)	*0.0102	*0.05

C) MFI By cervical length and pregnancy outcome

	Marker	Normal	Short	Mann Whitney	P. adj	TU	PT	Mann-Whitney	P. adj
Peripheral neutrophils	CD10	21242 (16689-25748)	21518 (17507-27745)	0.5696	0.8822	21702 (17261-26120)	14860 (12724-19887)	*0.0146	0.2000
	CD11b	17994 (13550-25726)	18903 (13630-26546)	0.7831	0.9380	18084 (13614-26694)	17212 (7685-18054)	0.1488	0.3900
	CD16	132757 (92375-219472)	129796 (81006-251125)	0.7509	0.5767	137216 (92615-227119)	56906 (51057-251125)	*0.0403	0.3435
	CD33	4331 (2862-5837)	4833 (3363-7313)	0.1579	0.5540	4331 (2998-6222)	4573 (2237-8128)	0.7651	0.9140
	CD35	22183 (18632-27241)	23457 (20247-27352)	0.6217	0.8822	22145 (18313-27339)	22687 (20301-30233)	0.387	0.6757
	CD55	64308 (51214-74556)	72501 (55040-81266)	0.1005	0.4633	65532 (53994-77957)	66612 (40119-82160)	0.9213	0.9140
	CD62l	28602 (17546-37649)	21573 (14215-27631)	*0.0151	0.1270	25816 (16537-35108)	21552 (13288-46880)	0.7182	0.8563
	CD63	7354 (4696-10440)	8977 (6123-10663)	0.1833	0.5450	7629 (4710-10419)	9196 (6530-14452)	0.2267	0.3900
	CD66b	15175 (11253-21657)	17332 (13490-19309)	0.6111	0.8822	15405 (12186-20141)	17084 (12612-25448)	0.6666	0.6757
	CD88	3822 (2958-5226)	5447 (3724-6864)	**0.0042	0.0985	4423 (3170-5878)	4548 (3619-7054)	0.4179	0.6460
Cervical neutrophils	CD10	17411 (11139-35229)	17441 (14027-27077)	0.9957	0.9530	17261 (11472-31185)	17896 (16125-28350)	0.7143	0.5657
	CD11b	7139 (3876-13552)	7266 (3703-11923)	0.7105	0.8686	7431 (3897-13278)	6051 (2945-11333)	0.2678	0.5017
	CD16	23006 (13987-34471)	19431 (12336-35096)	0.6173	0.9530	21308 (13655-36881)	21679 (13406-29805)	0.7159	0.6690
	CD33	3733 (2683-4862)	3636 (3150-6544)	0.438	0.8686	3567 (2705-4775)	5696 (3612-8092)	*0.0285	0.2430
	CD35	5599 (3524-16258)	3363 (2778-9676)	0.0546	0.1640	5234 (3303-15885)	3539 (2843-6940)	0.1568	0.2875
	CD55	61013 (50683-75415)	73895 (61013-114083)	*0.0281	0.2280	64713 (52649-78008)	104919 (55621-168737)	0.0814	0.4000
	CD62l	2575 (1822-5206)	1848 (1625-2690)	*0.0182	0.2280	2502 (1758-4758)	1975 (1578-2470)	0.0756	0.4000
	CD63	14510 (8541-44345)	16124 (9256-51767)	0.4077	0.8686	14525 (8557-41697)	16124 (12490-193870)	0.1838	0.5017
	CD66b	12479 (5348-63515)	16754 (6924-58781)	0.7338	0.9350	13332 (5493-59474)	23010 (6374-60516)	0.7207	0.6690
	CD88	19540 (4940-40746)	22481 (7446-43935)	0.5471	0.8686	20039 (6186-40096)	22481 (6699-52252)	0.4526	0.5775

Table S6. Statistical analysis of surface markers in peripheral and cervical neutrophils

A. Wilcoxon matched-pairs signed rank test of MFI between matched peripheral and cervical neutrophils. **B.** Differences between MFI depending on microbial composition were assessed with Kruskal-Wallis. **C.** Differences between MFI depending on cervical length and pregnancy outcome were assessed with Mann-Whitney. P value summary: * p < 0.05; **** p <0.0001

Figure S6. Matrix metalloproteinases

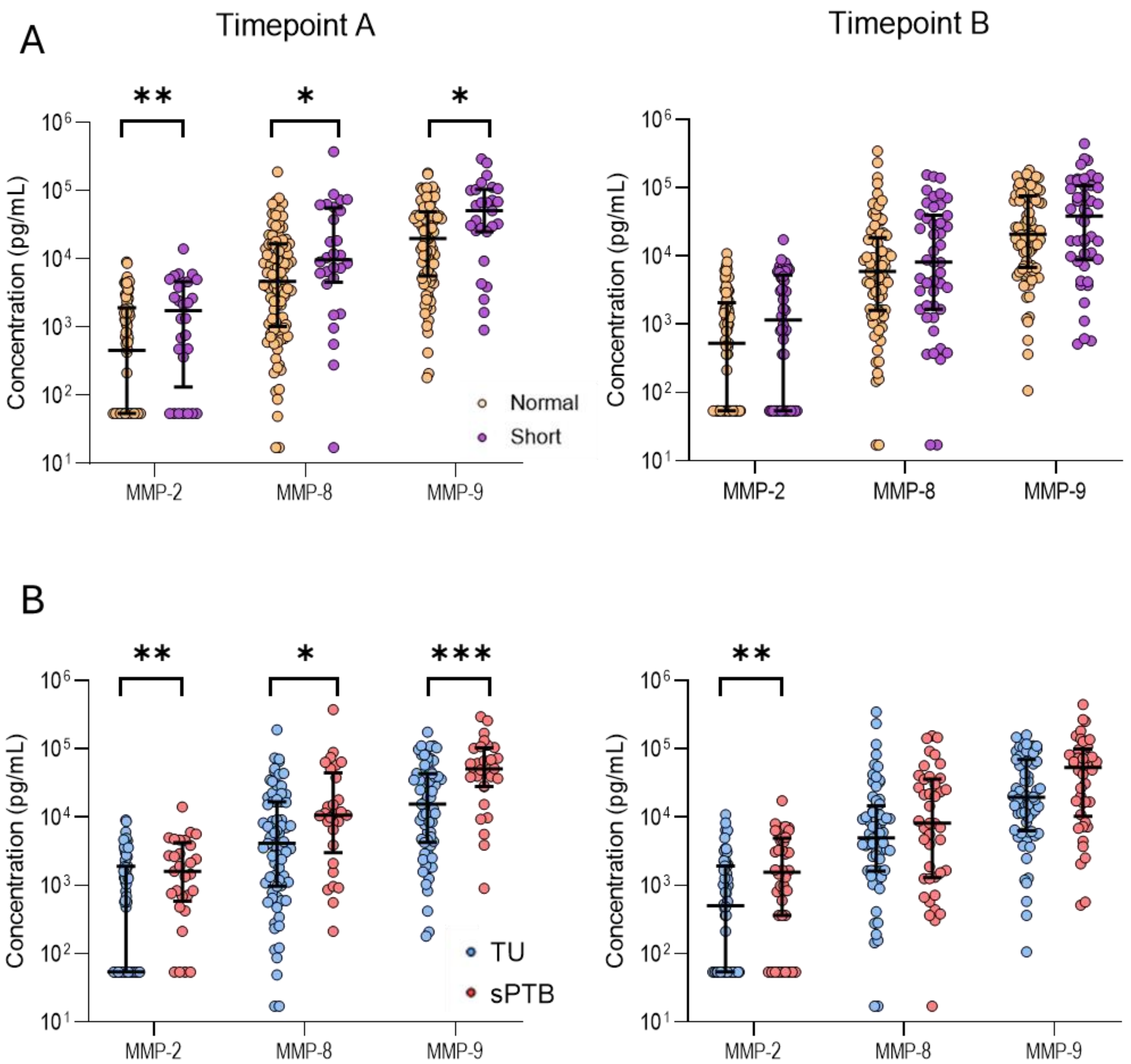

Figure S6. Cervicovaginal fluid matrix metalloproteinases concentrations according to cervical length and pregnancy outcome

A. Matrix metalloproteinase concentrations are presented in women who maintained a normal cervical length and those who developed a short cervix at Timepoint A and B. **B.** Matrix metalloproteinase concentrations are presented in women who delivered at term and who delivered spontaneously preterm at Timepoint A and B. Timepoint A: 12^{+0}-16^{+6} weeks of gestation. B: 20^{+0}-24^{+6} weeks of gestation. Median and interquartile ranges of MMP-2, MMP-8, MMP-9 (all, pg/mL). P value summary: * p < 0.05; ** p < 0.01; *** p <0.001

Table S7. Neutrophil surface markers

Antibody	Clone	Volume	Source	Role
PerCP/Cyanine5.5 anti-human CD45	2D1	1.7μL	BioLegend	Common leukocyte marker
Brilliant Violet 785™ anti-human CD15 (SSEA-1)	W6D3	0.5μL	BioLegend	Neutrophil identification Involved in cell-cell interactions, phagocytosis, stimulation of degranulation, and respiratory burst
PerCP-eFluor™ 710 anti-human CD14	61D3	0.5μL	eBioscience™, Invitrogen™	Monocyte marker, negative selection
eFluor™ 450 anti-human CD16	CB16	0.5μL	eBioscience™, Invitrogen™	Mature granulocytes, involved in degranulation
APC anti-human CD63	H5C6	0.5μL	BioLegend	Release of azurophilic granules
PE/Dazzle™ 594 anti-human CD66b	G10F	0.5μL	BioLegend	Respiratory burst, adhesion molecule, present in the membrane of specific granules
BD Horizon™ BV480 Mouse Anti-Human CD62L	DREG-56	0.5μL	BDBiosciences	L-selectin, implicated in tethering and rolling of leukocytes on endothelial surfaces and cell-cell interaction
Alexa Fluor® 700 anti-human CD11b	ICRF44	0.5μL	BioLegend	Integrin, cell-cell, and cell-extracellular matrix interactions; Intracellular storage pool of granules
APC/Cyanine7 anti-human CD10	HI10a	0.5μL	BioLegend	Neutral endopeptidase
Alexa Fluor® 647 anti-human CD33	P67.6	0.5μL	BioLegend	Immature neutrophils, adhesion molecule
BD OptiBuild™ BV510 Mouse Anti-Human CD55	IA10	0.5μL	BDBiosciences	Complement decay accelerating factor accelerates decay of C3 convertases
FITC anti-human CD35	E11	0.5μL	BioLegend	Complement receptor 1, CR1, C3b and C4b receptor, immune adherence receptor
BD OptiBuild™ BV421 Anti-Human CD88	D53-1473	0.5μL	BDBiosciences	Complement receptor, C5a receptor

Cell surface markers were selected according to its biologic role previously described by Elghetany et al. (104) and Ssemaganda et al. (105).
